# Unveiling the Formation and Evolution of the Cathode–Electrolyte Interphase in Lithium–Sulfur Batteries

**DOI:** 10.1002/advs.202518282

**Published:** 2025-12-23

**Authors:** Murilo Machado Amaral, Otavio Jovino Marques, André de Navarro de Miranda, Aline Carlos Oliveira, Gustavo Doubek, Gurpreet Singh, Hudson Zanin, Renato Garcia Freitas, Johanna Nelson Weker, Pablo Sebastian Fernandez

**Affiliations:** ^1^ Advanced Energy Storage Division Center for Innovation on New Energies School of Electrical Engineering and Computer University of Campinas (UNICAMP) Campinas SP 13083‐852 Brazil; ^2^ Mechanical and Nuclear Engineering Department Kansas State University Manhattan KS 66506 USA; ^3^ Stanford Synchrotron Radiation Lightsource SLAC National Accelerator Laboratory Menlo Park CA 94025 USA; ^4^ Advanced Energy Storage Division Center for Innovation on New Energies (CINE) Laboratory of Advanced Batteries School of Chemical Engineering University of Campinas (UNICAMP) Campinas SP 13083‐852 Brazil; ^5^ Institute of Physics & Institute of Chemistry Laboratory of Computational Materials Federal University of Mato Grosso Cuiabá MT 78060‐900 Brazil; ^6^ Center for Innovation on New Energies and Chemistry Institute University of Campinas (UNICAMP) Campinas SP 13083‐852 Brazil

**Keywords:** electrochemical impedance spectroscopy, in situ FTIR, lithium–sulfur batteries, X‐ray absorption spectroscopy

## Abstract

Lithium–sulfur batteries (LSBs) present high theoretical energy density, but their commercial viability is limited due to poor cyclability, primarily resulting from the shuttle effect. The formation and stability of the cathode–electrolyte interphase (CEI) are crucial for suppressing the shuttle effect and enhancing electrochemical reversibility. However, the CEI formation and evolution mechanism in LSBs remains unexplored. In this context, herein, the first real‐time investigation of the CEI using background‐subtracted in situ Fourier‐transform infrared (FTIR) spectroscopy is presented. The resulting FTIR spectra directly revealed electrolyte species consumption and reduced species’ emergence. Although these findings do not provide detailed compositional information about the CEI, the in situ FTIR results enabled direct tracking of electrolyte species undergoing decomposition associated with the formation and subsequent regeneration of the CEI. The in situ FTIR results are complemented by electrochemical impedance spectroscopy (EIS) measurements at different states of charge (SOCs), which indicate variations associated with structural changes in the interface layers. X‐ray absorption spectroscopy (XAS) at the sulfur K‐edge on post‐cycled electrodes confirmed the presence of sulfur species within the CEI. This work evidences the formation of the CEI, providing new mechanistic insights to support the design of electrodes and electrolytes for long‐term batteries.

## Graphical abstract

By integrating electrochemical impedance spectroscopy (EIS), background‐subtracted in situ Fourier‐transform infrared (FTIR) spectroscopy, and X‐ray absorption spectroscopy (XAS), this work reveals electrolyte decomposition and the reduced species involved in forming the cathode–electrolyte interphase (CEI) in lithium–sulfur batteries (LSBs), offering direct insight into CEI establishment and evolution.

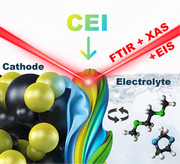

## 1 Introduction

Lithium–sulfur batteries (LSBs) are an attractive energy storage technology due to sulfur's high theoretical energy density (≈2600 Wh Kg^−1^), abundance, environmental benignity, and low cost.^[^ ^1^, ^2^ ^]^ However, the development of commercial LSBs is hindered by their poor cycling stability, which is attributed to several issues at both electrodes. The dissolution of lithium polysulfides (LiPSs), and their subsequent migration to the lithium anode is known as the shuttle effect, which causes irreversible material loss and capacity fade.^[^ ^3^, ^4^, ^5^ ^]^ Additional challenges of sulfur cathodes include sulfur's low electrical conductivity and the large volume expansion of sulfur particles upon conversion reactions (≈80%).^[^ ^6^ ^]^

Furthermore, the cathode–electrolyte interphase (CEI) ensures reversible electrochemical reactions and long‐term cycling stability. The CEI forms through electrolyte decomposition and acts as a passivation layer. However, the volume expansion of sulfur particles during cycling can cause cracks in the CEI, leading to the continuous consumption of electrolyte species for the regeneration of the CEI, which results in irreversible capacity fade.^[^ ^7^ ^]^ Additionally, the uneven lithium sulfide (Li_2_S) deposition on the cathode surface decreases sulfur utilization, leads to capacity fade, and increases interfacial impedance.^[^ ^8^ ^]^ The cathode structure influences the CEI, as porous host materials can promote the desolvation of lithium ions at the electrode interface, favoring the formation of a solidified CEI. A solidified CEI promotes the solvation of long‐chain intermediate LiPSs, mitigating sulfur loss and improving cycling stability.^[^ ^9^ ^]^ Moreover, a recent study conducted by Li et al.^[^ ^10^ ^]^ demonstrated that the CEI can be controlled by adjusting the cell voltage prior to cycling, promoting cycling stability to LSBs.

Among the techniques that can be used for studying interfacial processes, electrochemical impedance spectroscopy (EIS) is an interesting approach.^[^ ^11^, ^12^ ^]^ A recent study demonstrated that EIS can unveil individual resistance contributions from the solid‐electrolyte interphase (SEI), on the anode, and the CEI, on the cathode, providing a practical approach to monitor the stability of these layers.^[^ ^12^ ^]^ Nevertheless, EIS cannot directly provide structural information about the CEI.

Previous studies have employed in situ Fourier‐transform infrared (FTIR) spectroscopy on cathodes of LSBs primarily to monitor the presence of LiPSs.^[^ ^13^, ^14^, ^15^, ^16^ ^]^ Furthermore, a previous study monitored changes in the C─S vibration bond associated with LiPSs anchoring, promoted by the sulfur host.^[^ ^15^ ^]^ Another work conducted in situ FTIR spectroscopy to study the cathode of an LSB cell, and associated changes in the C─S vibration bond with the CEI.^[^ ^14^ ^]^ Nevertheless, these previous studies did not employ a *background‐subtraction* procedure, resulting in overlapping bands that obscured the identification of changes associated with electrolyte decomposition and the formation of new species.

Moreover, X‐ray absorption spectroscopy (XAS) measurements at the sulfur K‐edge can be used to investigate the composition of the interface layers of LSBs. Previous studies reported in the literature^[^ ^17^, ^18^ ^]^ indicated the presence of Li_2_SO_x_ species within the SEI formed on the lithium anode surface of LSBs. Therefore, based on its sensitivity to oxidation states, X‐ray absorption near‐edge structure (XANES) at the sulfur K‐edge can also be applied to monitor the chemical evolution of the CEI, providing insights into the formation of sulfur‐containing species within the CEI.

To address the gap in the study of the CEI formation and stabilization in LSBs, the present work combines EIS, background‐subtracted in situ FTIR spectroscopy, and XAS to provide complementary insights into the CEI. EIS measurements were conducted at different states of charge (SOCs), revealing insights into the resistance and the non‐ideal capacitive behavior of the interfacial layers. Furthermore, background‐subtracted in situ FTIR spectroscopy was conducted to track real‐time structural changes at the molecular level. According to the available literature, this is the first study that has monitored the formation, stability, and regeneration of the CEI using background‐subtracted in situ FTIR spectroscopy. The explicit mention of *background‐subtracted* is intentional, as previous studies have not followed this essential step, leading to spectral bands being overlapped by the strong signals from solvents and electrolytes. In addition, XAS measurements were conducted on post‐cycled electrodes at fully discharged and charged states to identify species that composed the CEI.

The electrochemical experiments were conducted using sulfur‐impregnated silicon oxycarbide (S@SiOC) as the cathode, selected for its stable cycling performance previously reported in a work conducted by Amaral et al.,^[^ ^19^ ^]^ which also included chemical and physicochemical characterizations for S@SiOC that confirmed the successful sulfur impregnation. The electrochemical experiments were conducted on a traditional electrolyte for LSBs, using lithium bis(trifluoromethanesulfonyl)imide (LiTFSI, LiN(SO_2_CF_3_)_2_) and lithium nitrate (LiNO_3_) as the salts, and 1,3‐dioxolane (DOL, CH_2_OCH_2_CH_2_O) and 1,2‐dimethoxyethane (DME, CH_3_OCH_2_CH_2_OCH_3_) as the solvents.

## 2 Experimental Section

### 2.1 Preparation of S@SiOC Electrodes

The SiOC material was prepared by the pyrolysis of 1,3,5‐trivinyl‐1,1,3,5,5‐pentamethyltrisiloxane (TPTS, Gelest, Inc., Pennsylvania, USA) at 800 °C under an argon flow, obtaining a ceramic derived from a polymeric precursor. The S@SiOC composite, composed of sulfur and SiOC at a mass ratio of 70:30 (w/w, S/SiOC), was prepared via a melt‐diffusion process. Subsequently, a slurry was prepared by mixing S@SiOC composite with polyvinylidene fluoride (PVDF, Alfa Aesar, USA) and acetylene carbon black (CB, Alfa Aesar, USA) in a mass ratio of 80:10:10 (w/w/w, S@SiOC/PVDF/CB), using N‐Methylpyrrolidone (NMP, Alfa Aesar, USA) as the solvent. The powders were homogenized using an agate mortar and pestle. The slurry was then cast onto an aluminum substrate using the doctor blade technique, following the procedure detailed in a previous study.^[^ ^19^ ^]^

### 2.2 Electrochemical Characterization of Coin Cells

Cyclic voltammetry (CV) measurements were conducted after the cell voltage stabilized, using a scan rate of 0.1 mV s^−1^ over a voltage window of 2.7–1.7 V vs Li^+^/Li^0^, except for the initial scan, which began at the open circuit voltage (OCV). All CV measurements were performed using the same two‐electrode CR2032‐type cell, comprising S@SiOC electrode as the cathode, lithium metal as the anode, and a glass fiber separator (Whatman, Inc., with *t* ≈ 260 µm). The electrolyte consisted of 1 mol dm^−3^ LiTFSI (Sigma–Aldrich) dissolved in a solvent mixture of DOL (Sigma‐Aldrich) and DME (Sigma‐Aldrich), in a ratio of 1:1 (v/v), containing 1 wt.% LiNO_3_ (Sigma–Aldrich). Potentiostatic EIS experiments were conducted using the same two‐electrode cell employed for CV experiments, using a single‐sine mode, a frequency range of 100 kHz–10 mHz, and a small sinusoidal signal of 10 mV (peak‐to‐peak) to linearize the impedance response. EIS was performed after five CV scans, ensuring a stable electrochemical condition of the electrode. The potentiostatic EIS was conducted at different SOCs of the cell, and the voltage values were obtained using linear sweep voltammetry (LSV). CVs and EIS experiments were performed using a Biologic SP‐200 potentiostat at 25 °C. Impedance data were analyzed using ZView software from Scribner Associates Inc.

To ensure consistent experimental conditions among EIS, FTIR, and XAS, all measurements were conducted using two‐electrode cells with the same configuration, employing S@SiOC as the cathode. EIS measurements were performed after five CV scans of a CR2032‐type cell, permitting the establishment of the SEI and the CEI. In contrast, in situ FTIR spectroscopy was conducted during the initial cycle of a fresh two‐electrode spectro‐electrochemical cell (SEC) to monitor the early‐stage formation and chemical evolution of the CEI, as well as the electrolyte decomposition. Furthermore, XAS measurements were conducted on post‐cycled S@SiOC cathodes extracted from CR2032‐type cells after 1 and 5 cycles, at the fully discharged and fully charged states. The XAS results provided complementary insights into the chemical composition of the CEI. Although each technique was conducted under different electrochemical conditions, the electrodes, the electrolyte, and the separator remained the same, ensuring consistency in the experiments and enabling a comprehensive investigation of the CEI formation and stabilization.

### 2.3 In Situ FTIR Spectroelectrochemistry

All FTIR measurements were performed using a Prestige‐21 spectrometer (Shimadzu Inc.) in the mid‐IR range (4000–400 cm^−1^), equipped with a liquid N_2_‐cooled mercury cadmium telluride (MCT) detector. The resulting spectra (composed by 128 interferograms) were plotted as absorbance, i.e., log_10_(R_0_/R), where R_0_ is the reference spectrum and R is the sample spectrum. The sample compartment has been purged with N_2_ gas, eliminating most atmospheric species (CO_2_ and H_2_O), thereby ensuring a measurement environment with a minimized contribution from atmospheric species and improving the signal‐to‐noise ratio.

The ex situ FTIR spectra of the solutions were acquired in the attenuated total reflectance (ATR) mode, and the solutions were placed directly onto a zinc selenide (ZnSe) ATR prism, positioned on top of a reflectance accessory (VeeMax II, Pike Technologies). The spectra of the solvents (i.e., DOL and DME) were taken using the sample compartment environment, purged with N_2_ gas, as the background. Moreover, the spectra of the salts (i.e., LiTFSI and LiNO_3_) were acquired using 7.0 mol dm^−3^ LiTFSI dissolved in DOL/DME (1:1, v/v, unsaturated) and 0.75 mol dm^−3^ LiNO_3_ dissolved in DOL/DME (1:1, v/v, saturated). The spectra of salts dissolved in the organic solvents were taken using a pure DOL/DME (1:1, v/v) solution as the background, aiming to directly identify the specific signals of the salts in the solution.

**Figure 1:**
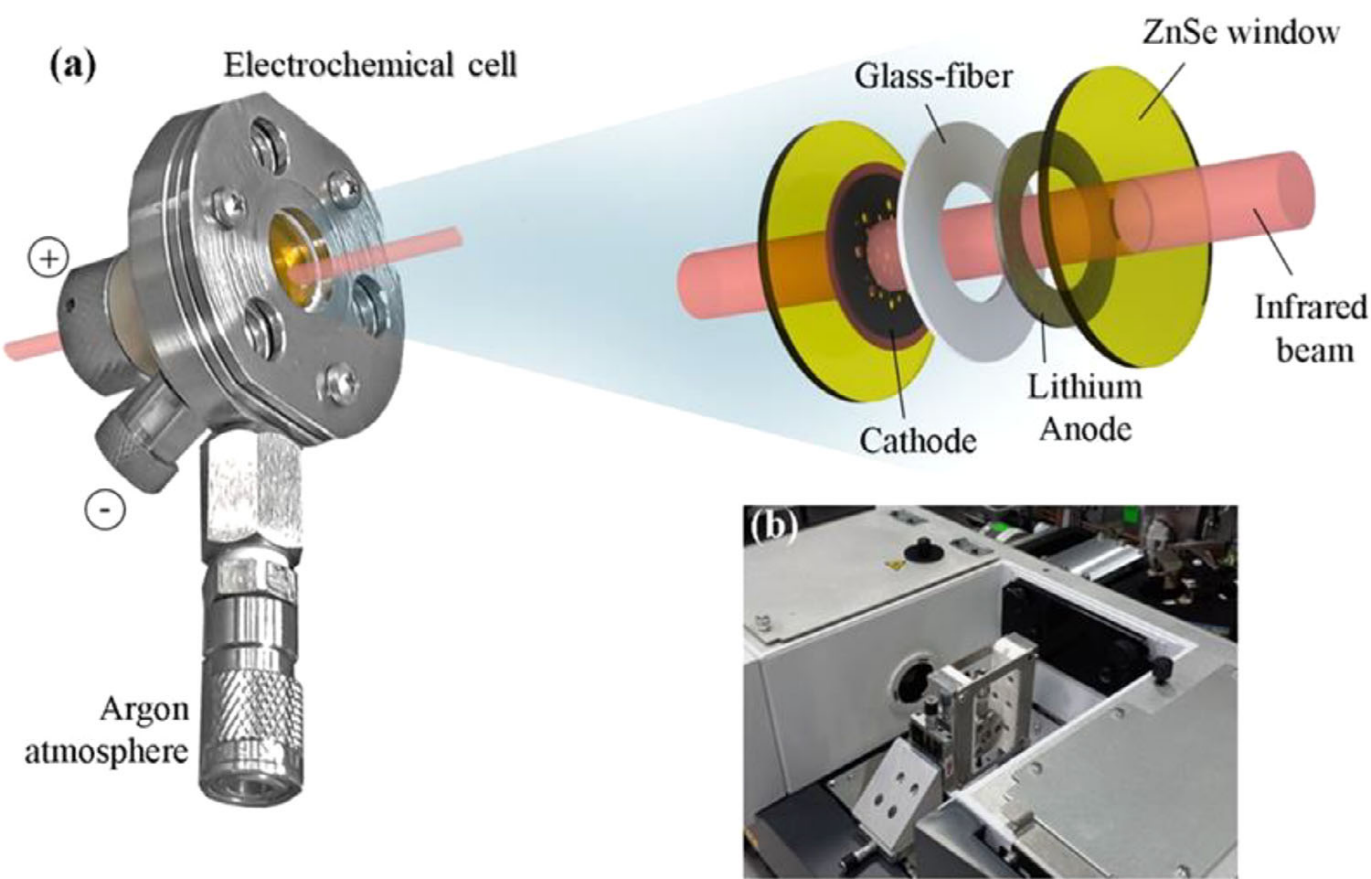
Apparatus of the in situ transmission IR SEC set‐up used to conduct the in situ measurements: a) SEC used for in situ IR spectroscopy, b) sample compartment of the FTIR spectrometer, containing the SEC positioned in the holder.

The in situ FTIR measurements were performed in transmittance mode, using a SEC equipped with two ZnSe optical windows, comprising an S@SiOC cathode, a lithium metal anode (Gelon Energy, Inc., with ø ≈ 15.6 mm, *t* ≈ 250 µm), and a glass‐fiber membrane (Whatman, Inc., with *t* ≈ 260 µm), as shown in **Figure** **1**a. The LSB cell was assembled inside an Ar‐filled glovebox (MBraun, Inc., with O_2_ < 0.1 ppm and H_2_O < 0.1 ppm), using 80 µL of the electrolyte. Furthermore, central holes were made in the glass fiber separator and the lithium anode, allowing the IR transmission through the cell. Several pinholes were previously made in the S@SiOC cathode, allowing the IR beam to pass through them after being partially absorbed by the sulfur cathode and providing structural information about the CEI under operating conditions. The cell was placed into a holder with vertical adjustment to precisely align its position with the IR beam, as shown in Figure 1b.

Two electrolytes were used for the in situ FTIR spectroscopy measurements. The first consisted of 1.0 mol dm^−3^ LiTFSI dissolved in DOL/DME (1:1, v/v) containing 1 wt.% LiNO_3_ (Sigma–Aldrich). The second electrolyte was an additive‐free electrolyte, comprising 1.0 mol dm^−3^ LiTFSI dissolved in DOL/DME (1:1, v/v), referred to as *LiNO_3_‐free electrolyte*. The spectra were acquired during a galvanostatic charge‐discharge (GCD) process, conducted between 2.7 and 1.7 V vs Li^+^/Li^0^, at a C‐rate of 0.2 C (considering 1 C = 1675 mA g_s_^−1^), using a PGSTAT 204 potentiostat (Autolab Inc.). A background spectrum was taken prior to the discharge and charge processes, allowing the observation of the species being consumed or formed during each process. The spectra plotted for the in situ measurements were vertically offset to avoid overlapping and highlight the emergence of upward and downward bands.

### 2.4 X‐Ray Absorption Spectroscopy

XAS measurements were conducted at beamline 4‐3 of the Stanford Synchrotron Radiation Lightsource (SSRL) at SLAC National Accelerator Laboratory. The measurements were performed in fluorescence mode using a passivated implanted planar silicon (PIPS) semiconductor detector, at the sulfur K‐edge, and a beam spot size of 1 mm × 7 mm. The samples were placed inside a helium (He)‐filled box during the measurements, and the X‐ray energy was calibrated using sodium thiosulfate (Na_2_S_2_O_3_, Sigma Aldrich).

The measurements were performed on pristine and post‐cycled S@SiOC electrodes. For the XAS measurements of the post‐cycled S@SiOC electrodes, CR2032‐type coin cells were assembled and cycled at a gravimetric current of 167.5 mA g_s_^−1^. After cycling, the cells were transferred into an Ar‐filled glove box (Vacuum Atmospheres Co.), where they were decrimped, and the electrodes were extracted. The electrodes were then rinsed with dimethyl carbonate (DMC) and dried overnight at 80 °C on a hot plate. Next, the electrodes were placed on a sample holder, sealed on both sides with polymeric tape, placed inside an aluminized pouch, and transferred to the beamline for XAS measurements. The spectra were analyzed with the Athena software package. Spectra from post‐cycled electrodes were normalized to the spectrum of the pristine electrode.

Reference XAS measurements were conducted for commercial sulfur powder (S_8_, Sigma–Aldrich) and LiTFSI salt (Sigma–Aldrich). An additional reference measurement was performed by soaking a glass fiber membrane with an electrolyte solution comprising 1.0 mol dm^−3^ LiTFSI in DOL/DME (1:1, v/v) containing 1 wt.% LiNO_3_ as an additive. After drying, the membrane was placed in a sample holder inside an Ar‐filled glove box and transferred to the beamline.

## 3 Results and Discussion

### 3.1 Electrochemical Characterization

EIS measurements were conducted at various SOCs to investigate electrochemical processes within the LSB cell, including interfacial phenomena (e.g., CEI) and redox reactions. These measurements were conducted on two‐electrode CR2032‐type LSB cells, comprising S@SiOC as the cathode. The electrochemical performance of the S@SiOC cathode was previously reported by Amaral et al.^[^ ^19^ ^]^ In that study, the S@SiOC cathode exhibited stable cycling performance for 200 cycles, followed by moderate capacity fade. Therefore, S@SiOC was selected as a cathode for the present work to enable a multi‐technique investigation of the CEI under stable electrochemical conditions.

The EIS measurements were performed after five CVs (Figure S1, Supporting Information), previously conducted to stabilize the cell's electrochemical behavior. The CV curves of the S@SiOC electrode exhibited only the characteristic sulfur redox peaks, indicating that the SiOC host did not promote additional redox reactions within the investigated voltage range.^[^ ^20^, ^21^, ^22^ ^]^ Moreover, a previous study conducted by Amaral et al.^[^ ^19^ ^]^ compared the cycling performance of S@SiOC with a *host‐free* electrode and demonstrated the superior cycling stability of the S@SiOC cathode. These previous electrochemical results confirmed the lower stability of a *host‐free* electrode, which would not ensure stable electrochemical conditions for studying the CEI.

Furthermore, EIS results can be interpreted using equivalent circuit (*eqvcrt*) models, indicating the processes occurring within a battery system. Different *eqvcrts* have been employed in the study of batteries.^[^ ^23^, ^24^ ^]^ In this work, the physical criterion for transitioning among the three *eqvcrts* (i.e., *eqvcrt‐i*, *eqvcrt‐ii*, and *eqvcrt‐iii*) was determined by changes observed in the Nyquist plots, including variations in their time constants (*τ*), and changes in the slope of the diffusion region. The variations in the SOC may substantially modify the electrochemical interface, thereby giving rise to distinct *eqvcrts* representations. In this work, the designed *eqvcrts* represent heuristic but physically grounded simplifications of the electrochemical system.

**Figure 2:**
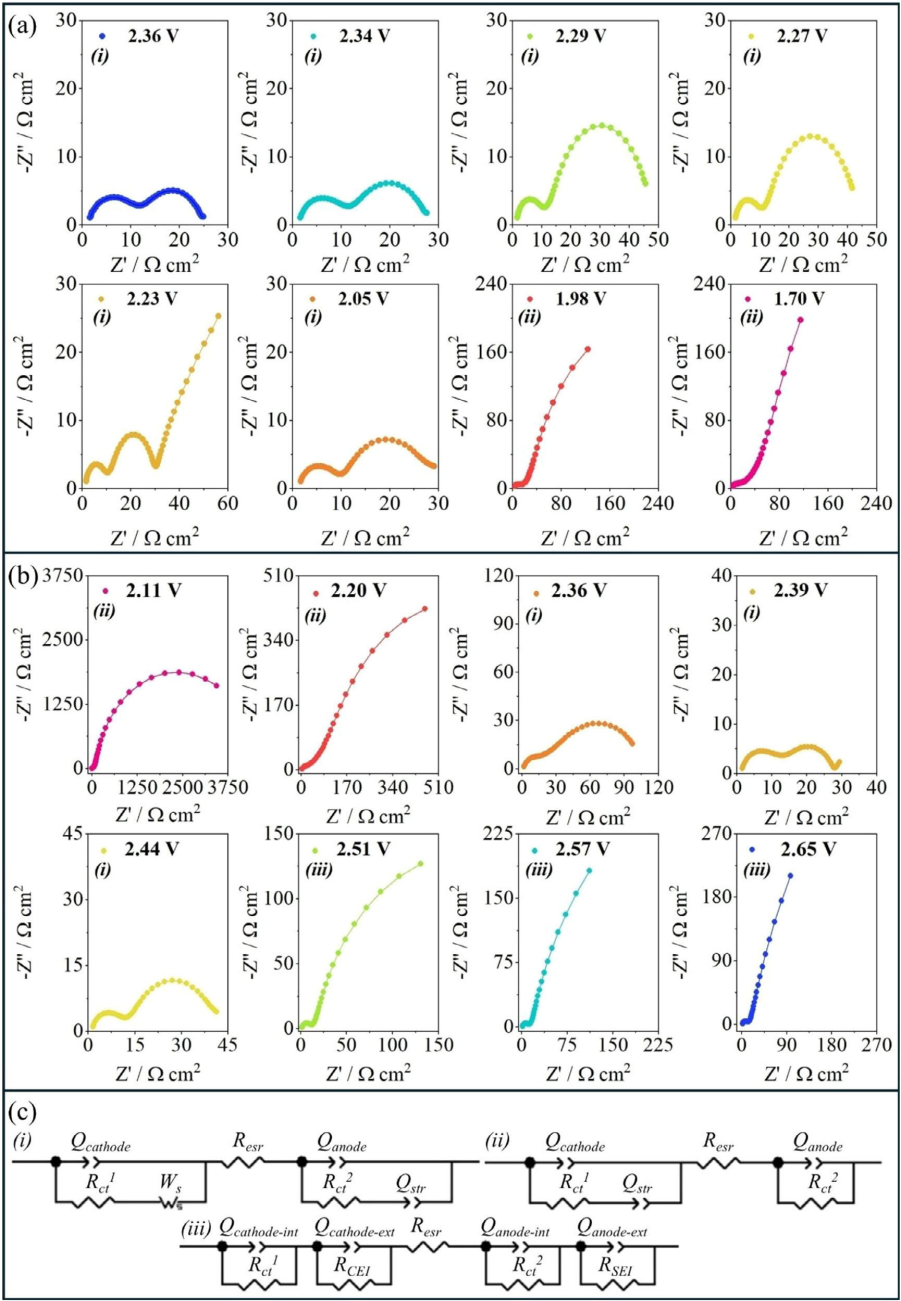
Nyquist plot obtained of an LSB for the a) negative and b) positive polarization, and the c) equivalent electrochemical circuits (*eqvcrts*) used for the fitting of EIS data. Positive electrode: S@SiOC. Negative electrode: lithium metal (Li^0^). Electrolyte: 1.0 mol dm^−3^ LiTFSI in DOL/DME (1:1, v/v) with 1 wt.% LiNO_3_. Measurements were performed using a two‐electrode CR2032‐type cell.

The initial EIS measurement was conducted at the OCV (i.e., SOC ≈ 100%), and the cell was further partially discharged, targeting specific voltage values using LSV, and EIS measurements were performed at different SOCs. The corresponding Nyquist plots resulting from EIS measurements are shown in **Figure** **2**a,b, attributed to the data obtained during negative and positive polarization, respectively.

Among the *eqvcrts* used for the EIS fitting, the *eqvcrt‐i* model comprised a finite‐length Warburg element with short‐circuit boundary conditions (*W_s_*), used to fit EIS data that showed diffusion impedance. The *eqvcrt‐ii* model does not comprise a Warburg element, providing a better fit to other EIS data. Furthermore, the *eqvcrt‐iii* model provided a deconvolution of the impedance into internal and external parameters, such as resistance associated with interfacial phenomena (i.e., SEI and CEI), applied at higher SOC values, where interfacial layers were more stable. In all *eqvcrts*, the capacitive response was represented by a constant phase element (CPE), which is used to describe the non‐ideal capacitive behavior,^[^ ^25^ ^]^ as detailed in Equation S1 (Supporting Information). The CPEs have been labeled as *Q* in the *eqvcrts* detailed in Figure 2ci–iii. The total impedance equations of the *eqvcrt‐i*, *eqvcrt‐ii*, and *eqvcrt‐iii* models are provided in Equations S2–S4 (Supporting Information), respectively.

During the negative polarization, Li^+^ diffusion occurred within the S@SiOC structure, resulting in the reduction of sulfur to soluble LiPSs, and subsequently to Li_2_S, along with changes in the electrode‐electrolyte interface layers (i.e., SEI and CEI), which were previously formed during the initial scans. During the negative polarization, the EIS measurements were conducted for the cell at the OCV (2.36 V vs Li^+^/Li^0^), and the cell was subsequently discharged until the voltage of 1.7 V vs Li^+^/Li^0^. The Nyquist plots resulting from EIS measurements obtained at voltages between 2.36 and 2.05 V vs Li^+^/Li^0^, during the negative polarization, displayed two semicircles corresponding to interfacial processes at the cathode and anode. The increase in the high‐frequency semicircle observed at the voltages of 2.29 and 2.27 V vs Li^+^/Li° can be attributed to structural changes in the interfacial layers. Moreover, the Nyquist plots at the voltages of 1.98 and 1.7 V vs Li^+^/Li^0^ displayed a depressed semicircle, which can be related to interfacial stabilization processes occurring on the electrodes’ surfaces.

Furthermore, a potentiostatic EIS measurement was performed at the fully discharged state (OCV of 2.11 V vs Li^+^/Li^0^). The cell was subsequently positively polarized via LSV, and EIS spectra were recorded at selected voltages up to 2.65 V vs Li^+^/Li^0^. During the positive polarization of the cell, the Nyquist plots at voltages of 2.11 and 2.2 V vs Li^+^/Li^0^ exhibited high impedance responses, with the low‐frequency semicircle being suppressed (see Figure 2b). Next, the increase in the semicircle displayed in the high frequencies of the Nyquist plots at voltages of 2.36, 2.39, and 2.44 V vs Li^+^/Li° can be attributed to structural changes in the interfacial layers (i.e., SEI and CEI). Moreover, the Nyquist plots at voltages of 2.51, 2.57, and 2.65 V vs Li^+^/Li^0^ exhibited a depressed semicircle, which can be attributed to the stabilization of the interfacial layers.

The charge transfer process is described as *Z_ct_*, which is an impedance that generally consists of a charge‐transfer resistance (*R*_ct_) in parallel with a CPE. In the *eqvcrts*, *Q*_cathode_ represents the non‐ideal capacitive response associated with the anode, including the CEI. Furthermore, *R*_esr_ is the equivalent series resistance, *Q*_anode_ consists of the non‐ideal capacitive response associated with the anode, including the SEI. The *eqvcrt‐i* and *eqvcrt‐ii* comprised a CPE referred to as *Q*_str_, which is associated with non‐ideal capacitive behavior at the S@SiOC electrode surface, particularly related to capacitive contributions of the SiOC host. The fitting results for the EIS data displayed in Figure 2a,b are detailed in Table S1 (Supporting Information). The impedance data were validated by Kramers–Kronig tests, which indicated low chi‐square (ca. χ^2^ < 10^−5^) and sum of squares values (ca. SS < 10^−3^), as shown in Table S2 (Supporting Information).

These SEI and the CEI behave as passivating films and are formed via electrolyte decomposition, acting as an ionic conductive and electron‐insulating layer,^[^ ^26^ ^]^ influencing the charge‐transfer kinetics of lithium batteries.^[^ ^27^ ^]^ Thus, the establishment of the SEI and CEI can be associated with changes in the values of *τ*. Furthermore, evaluating the *τ* is essential, as it indicates how rapidly a system provides an electrochemical response, and can offer insight into different electrochemical processes.^[^ ^28^ ^]^ The values of *τ* were determined by Equation (1), where *R* is the resistance, *Q* is the magnitude of the CPE, and *n* is a real number between 0 and 1 known as the CPE exponent, as reported in several previous works.^[^ ^29^ ^,^ ^30^ ^]^

(1) $$\tau ={\left(R\times Q\right)}^{1/n}$$

**Figure 3:**
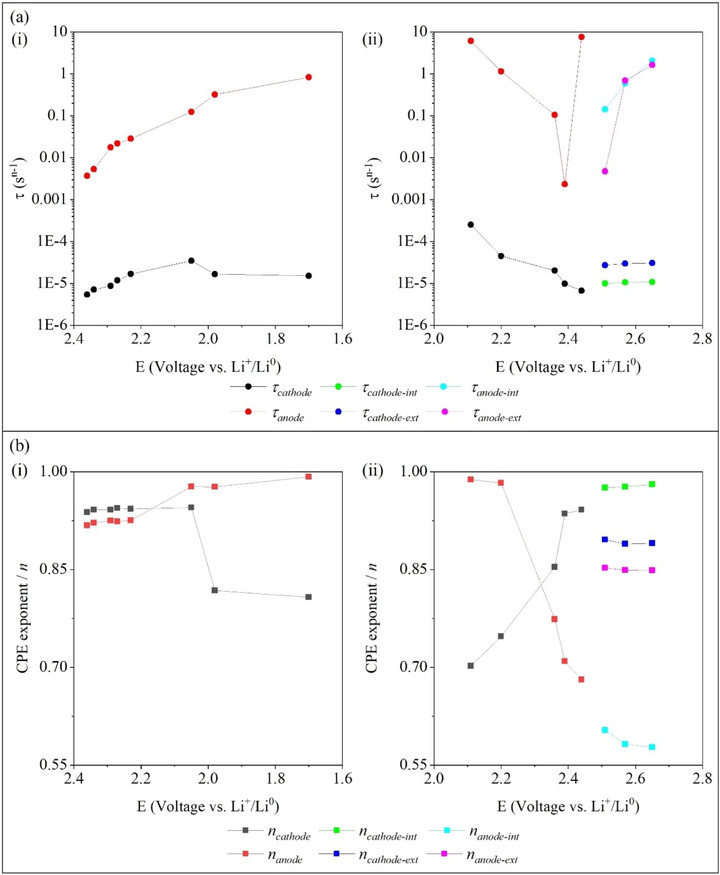
Evolution of the a) *τ* and b) *n* values extracted from the EIS fitting, as a function of the voltage, during the (i) discharge and (ii) charge of the LSB cell. The values of *τ* were plotted on a logarithmic scale.

**Figure** **3**a shows the *τ* values for the cathode (*τ*_cathode_) and the anode (*τ*_anode_), extracted from the EIS fitting, particularly during the negative and positive polarization, as shown in Figure 3a‐i,a‐ii, respectively. In summary, the *τ*_anode_ showed values higher than the *τ*_cathode_ for all measurements, indicating that the lower *τ*_cathode_ value is associated with faster ionic or electronic dynamics, and possibly faster interfacial kinetics. During the negative polarization, in which lithium is extracted from the anode, the following trend was observed: *τ*_cathode_ < *τ*_anode_ (see Figure 3a‐i), and a similar trend was observed during the positive polarization (see Figure 3a‐ii). The variation in the values of the *τ*_cathode_ and *τ*_anode_ can possibly indicate stabilization and progressive regeneration of the interfacial layers.

In Figure 3a‐ii, at the voltages of 2.51, 2.57, and 2.65 V vs Li^+^/Li^0^, the *τ*_cathode_ and *τ*_anode_ were deconvoluted into internal (*τ*_int_ = *R_int_* ×*Q_int_*) and external (*τ*_ext_ = *R_ext_* ×*Q_ext_*). The *τ*_anode‐int_ and *τ*_anode‐ext_ increased during the positive polarization and showed comparable values at the voltages of 2.57 and 2.65 V vs Li^+^/Li^0^. Furthermore, the *τ* attributed to the cathode showed the following trend: *τ*_cathode‐int_ < *τ*_cathode‐ext_, which can be attributed to the stabilization of CEI.

The CPE exponent (*n*) of the anode (*n*_anode_) increased during the negative polarization (Figure 3b‐i), and decreased during the positive polarization (see Figure 3b‐ii), likely due to a partial dissolution or regeneration of the SEI. Otherwise, the *n* of the cathode (*n*_cathode_) decreased during the negative polarization (Figure 3b‐i) and increased during the positive polarization (Figure 3b‐ii), indicating an opposite trend compared to the *n*_anode_. Thus, the opposite trend likely indicates asymmetric capacitive response for the cathode and the anode, likely arising from structural changes of the SEI and the CEI. It is essential to observe that the SEI and CEI were already formed before the EIS measurements, as CVs were previously performed (Figure S1, Supporting Information).

For the EIS measurements conducted at 2.51, 2.57, and 2.65 V, the *n_cathode_* and *n_anode_* were deconvoluted into internal and external values. The *n* values showed a nearly stable behavior for these voltages, following the trend: *n_cathode‐int_ > n_cathode‐ext_ > n_anode‐ext_ > n_anode‐int_*. The higher *n* values for the cathode indicate that it exhibits a more ideal capacitive behavior than the anode. The nearly stable trend observed for the *n* values at the voltages of 2.51, 2.57, and 2.65 V likely indicates that the SEI and CEI were stable at these voltages.

In summary, the EIS results indicated the stabilization of the SEI and CEI, governed by the applied voltage and stability of the electrolyte components. The EIS analysis revealed changes in the CEI under polarization conditions based on variations observed in the *τ* and the *n*. However, as EIS is a model‐dependent technique, background‐subtracted in situ FTIR spectroscopy was performed to get direct molecular‐level evidence of the CEI formation and establishment (Section 3.2).

### 3.2 Spectro‐Electrochemical Characterization of the Cathode–Electrolyte Interphase by Background‐Subtracted In Situ FTIR Spectroscopy

Background‐subtracted in situ FTIR spectroscopy was performed to monitor structural changes resulting from the electrolyte decomposition and the formation of chemical species constituting the CEI. However, assigning specific vibration bands observed in the resulting spectra is challenging, as the characteristic bands of organic electrolytes can often overlap additional bands attributed to species formed under operating conditions. Therefore, a background spectrum was taken when the cell was at the OCV, before starting the electrochemical measurements, which was subtracted from the subsequent spectra during the electrochemical measurements. This approach is referred to as a *background‐subtraction* procedure, which enables direct tracking of the consumption and emergence of species.

**Figure 4:**
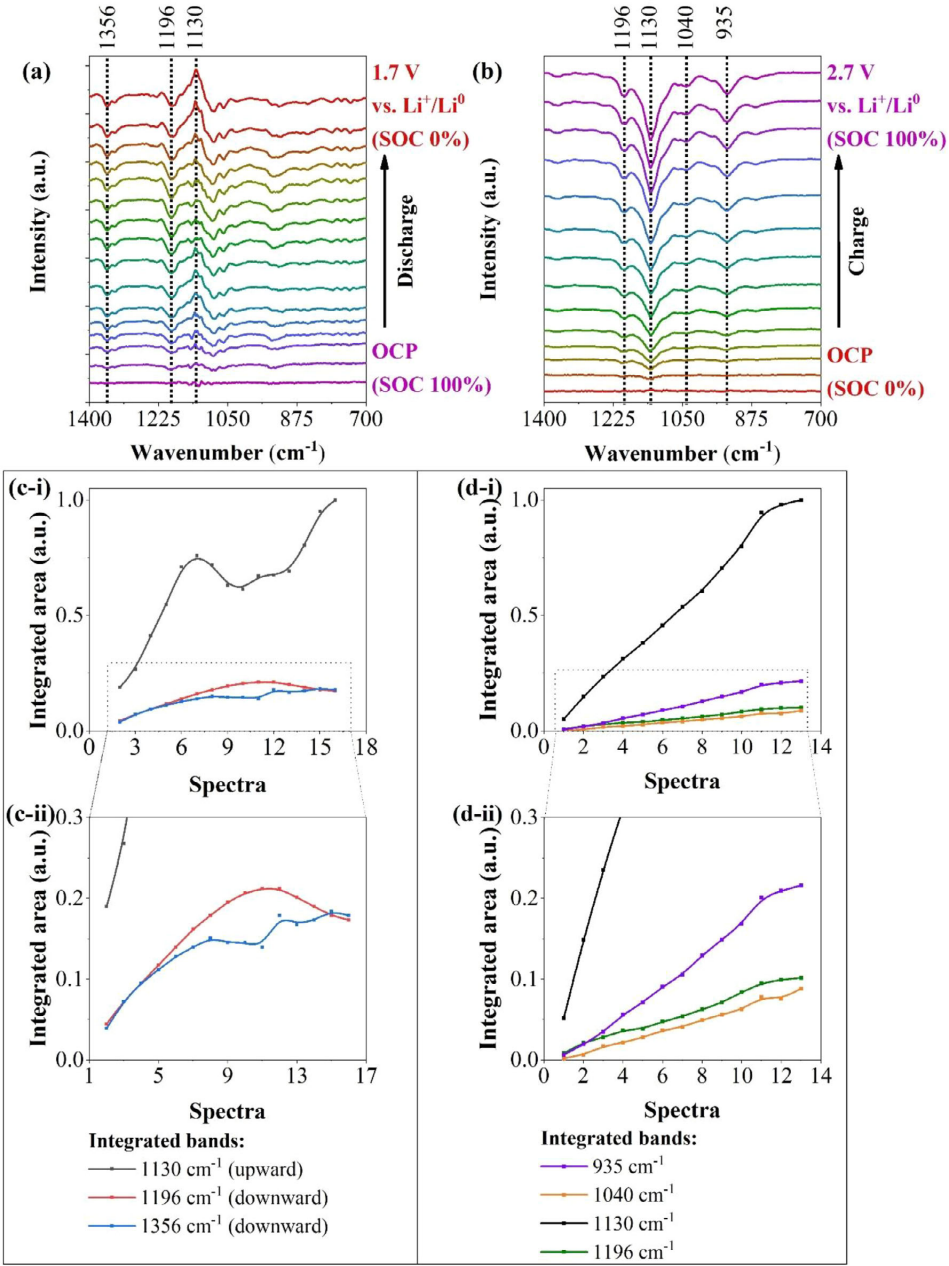
In situ FTIR difference spectra of the LSB cell containing 1.0 mol dm^−3^ LiTFSI in DOL/DME (1:1, v/v) with 1 wt.% LiNO_3_ as the electrolyte during the initial a) discharge and b) charge, displaying the consumption of electrolyte species (downward bands) and the formation of reduced species (upward bands) that possibly composed the CEI. Integrated areas of the most intensive bands displayed during the c) discharge and d) charge. Cathode: S@SiOC. Anode: lithium metal (Li^0^).

**Figure** **4** shows the spectra obtained during the discharge, starting from the OCV, ≈2.36 V vs Li^+^/Li^0^, and ending at 1.7 V vs Li^+^/Li^0^ (see the GCD curves in Figure S2, Supporting Information). The resulting spectra displayed the presence of downward bands (indicating a reduction in intensity at those wavenumbers), and the most intense bands were positioned at 1356 and 1196 cm^−1^ (Figure 4a). During the charging process, downward bands were observed at 1196, 1040, and 935 cm^−1^ (Figure 4b). These downward bands mentioned are associated with the decomposition of electrolyte salts and solvents (Figure S3 and Table S3, Supporting Information). To identify the solvents and salts consumed during the initial discharge, the last spectrum obtained during the discharge of the cell was compared to reference measurements conducted under the same conditions (see Figure S3, Supporting Information). These reference measurements consisted of ex situ FTIR spectra of the individual solvents and salts used in the electrolyte.

Moreover, during the discharge process, the spectra displayed an upward band at 1130 cm^−1^ (Figure 4a), which is associated with the formation of new species, likely including those that constitute the CEI. Furthermore, during the charge process, the spectra displayed a downward band at 1130 cm^−1^ (Figure 4b), showing a similar shape to the upward band previously observed at 1130 cm^−1^ during the discharge process (Figure 4a). The similarities in the shape of these upward and downward bands, positioned at 1130 cm^−1^, during the discharge and charge processes indicate that part of the species formed during the discharge was decomposed during the charge process. Thus, this result possibly indicates the partial dissolution of the CEI during charging. Nevertheless, the charge process also revealed the presence of downward bands attributed to electrolyte decomposition (Figure 4b; Figure S3, Supporting Information), which can likely be associated with the consumption of electrolyte species for the regeneration of the CEI.

The most intense bands observed during the discharge and chargewere integrated to quantitatively analyze their behavior. The bands integrated during the discharge exhibited different trends, indicating that they were attributed to different species, and formed or consumed at different rates (see Figure 4c). In contrast, the integrated downward bands displayed during the charging process followed a similar trend, indicating that the bands likely originate from the same sources and were consumed at the same rate (see Figure 4d).

Although the in situ FTIR spectra did not provide a clear identification of the reduced species formed in the initial discharge, previous studies have investigated the reaction mechanisms and reduced species formed during electrolyte decomposition, including DOL and DME solvents, as well as LiTFSI and LiNO_3_ salts. Furthermore, the reduction of DOL has been investigated in a previous work conducted by Aurbach et al.^[^ ^31^ ^]^, indicating the formation of species such as lithium formate (HCO_2_Li) and lithium alkoxide species (ROLi),^[^ ^31^ ^]^ where R is an alkyl group.^[^ ^32^ ^]^ Another study conducted by Aurbach and Granot^[^ ^33^ ^]^ proposed a reduction mechanism for DME solvent, which indicated the formation of lithium methoxide (LiOCH_3_) and CH_3_OCH_2_CH_2_OLi.

The decomposition of LiTFSI salt has also been previously studied, as demonstrated in a study conducted by Aurbach et al.,^[^ ^34^ ^]^ which proposed possible reaction mechanisms for the LiTFSI reduction, involving the formation of reduced species such as Li_2_S_2_O_4_, Li_2_S, and Li_2_SO_3_, as detailed in Equations (2)–(4).

(2) $$\mathrm{LiN}{\left({\mathrm{SO}}_{2}{\mathrm{CF}}_{3}\right)}_{2}+ne^{-}+\mathrm{nL}i^{+}\to {\mathrm{Li}}_{3}N+{\mathrm{Li}}_{2}S_{2}O_{4}+\mathrm{LiF}+C_{2}F_{x}{\mathrm{Li}}_{y}$$

(3) $${\mathrm{Li}}_{2}S_{2}O_{4}+6e^{-}+\;6{\mathrm{Li}}^{+}\to 2{\mathrm{Li}}_{2}S+4{\mathrm{Li}}_{2}O$$

(4) $${\mathrm{Li}}_{2}S_{2}O_{4}+4e^{-}+\;4{\mathrm{Li}}^{+}\to {\mathrm{Li}}_{2}{\mathrm{SO}}_{3}+{\mathrm{Li}}_{2}S+{\mathrm{Li}}_{2}O$$

Furthermore, a previous study conducted by Zhao et al.^[^ ^35^ ^]^ proposed a reaction mechanism for the LiNO_3_ salt decomposition, indicating the formation of Li_2_O and NO_2_, followed by the subsequent formation of SO_3_, as detailed in Equations (5) and (6).

(5) $$\mathrm{LiN}O_{3}+{\mathrm{Li}}^{+}+e^{-}\to {\mathrm{Li}}_{2}O\;+\;{\mathrm{NO}}_{2}$$

(6) $$15{\mathrm{Li}}_{2}O+22{\mathrm{NO}}_{2}+{S_{5}}^{2-}\to 22{{\mathrm{NO}}_{2}}^{-}+5{{\mathrm{SO}}_{3}}^{2-}+30{\mathrm{Li}}^{+}$$

**Figure 5:**
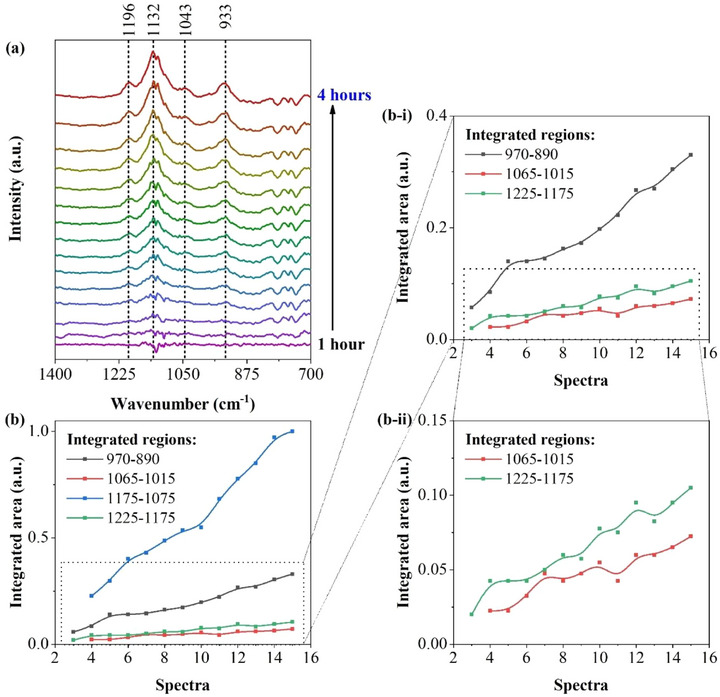
a) FTIR difference spectra of the LSB cell containing 1.0 mol dm^−3^ LiTFSI in DOL/DME (1:1, v/v) with 1 wt.% LiNO_3_ as the electrolyte under the OCV, for 4 h, showing the adsorption of electrolyte species on the cathode surface and the pre‐formation of the CEI. b) Integrated areas of the bands formed during the in situ FTIR measurement. Cathode: S@SiOC. Anode: lithium metal (Li^0^).

Moreover, the LSB system was also investigated by background‐subtracted FTIR spectroscopy without polarization conditions, using the same SEC. In this experiment, the cell rested for ≈4 h at the OCV, and the resulting spectra revealed four upward vibration bands at 1196, 1132, 1043, and 933 cm^−1^ (Figure **5**a). These bands are likely associated with the species resulting from spontaneous reactions between soluble LiPSs and electrolyte species, which can also contribute to a partial self‐discharge, as previously reported.^[^ ^36^ ^]^ The upward bands observed in the resulting spectra (Figure 5a) increased at a similar rate (Figure 5b), indicating that the species may have originated from common precursors and were formed at the same rate. The absence of downward bands in these spectra indicates that electrolyte species were not being consumed, unlike the experiment conducted when the LSB cell was being discharged and charged (see Figure 4).

In situ FTIR measurements were also conducted in an SEC comprising 1.0 mol dm^−3^ LiTFSI in DOL/DME (1:1, v/v) as the electrolyte (i.e., *LiNO_3_‐free*) to evaluate the contribution of LiNO_3_ to CEI formation (the GCD curve is shown in Figure S4, Supporting Information). The spectra revealed the appearance of two upward bands associated with the formation of reduced species, located at 1211 and 1134 cm^−1^. The band positioned at 1134 cm^−1^ was the most intense and was also observed in the in situ FTIR spectra of the cell comprising the *LiNO_3_‐free* electrolyte (Figure S5a, Supporting Information). However, this band appeared significantly sharper than the one observed at 1130 cm^−1^ in the spectra of the cell containing LiNO_3_ additive (Figure 4). This indicates that the LiNO_3_ additive promoted the formation of additional reduced species, resulting in the presence of a broader band. Furthermore, two bands associated with the electrolyte decomposition were observed in the in situ FTIR spectra of the LiNO_3_‐free system, positioned at 1354 and 1057 cm^−1^ (Figure S5a, Supporting Information).

During the charging process, electrolyte decomposition was also observed. In contrast, the bands attributed to the formation of reduced species showed a reverse behavior upon charging process (i.e., from upward to downward), indicating that the CEI underwent partial dissolution while charging the cell (Figure S5b, Supporting Information), similar to the resulting spectra observed in the cell containing the LiNO_3_ electrolyte additive (Figure 4). Thus, the continuous electrolyte decomposition observed during the charging process indicates the regeneration of the CEI. The integrated area of the band centered at 1134 cm^−1^, during the discharge, associated with the formation of electrolyte reduced species, showed an increasing rate similar to the band centered at 1354 cm^−1^ (Figure S5c, Supporting Information). This similar increasing rate is due to the decomposition of species present in the LiTFSI salt (see Figure S6, Supporting Information). This correlation indicates that LiTFSI is a primary precursor for CEI formation in the system comprising a *LiNO_3_‐free* electrolyte. In contrast, the band positioned at 1211 cm^−1^, attributed to the formation of reduced species, presented an increasing rate similar to the band at 1057 cm^−1^ (Figure S5c, Supporting Information).

During the charging process, the downward bands at 1194 and 1126 cm^−1^ likely indicate partial dissolution of the CEI (Figure S5b, Supporting Information), as spectra resulting from the discharge exhibited two upward bands at 1211 and 1134 cm^−1^, which are comparable positions (Figure S5a, Supporting Information). Furthermore, the spectra resulting from the charging process also displayed two additional downward bands, positioned at 1358 and 935 cm^−1^, assigned to electrolyte consumption (Figure S5b, Supporting Information). All downward bands displayed in the spectra resulting from the charging process displayed a similar trend (Figure S5d, Supporting Information). In summary, the decomposition of most bands resulted from LiTFSI, DOL, and DME (see Figure S6 and Table S4, Supporting Information).

To investigate the influence of the polarization in the SEC comprising the *LiNO_3_‐free* electrolyte, one measurement was performed while the cell was at the OCV for 4 h. The in situ FTIR results of the cell at the OCV did not show downward bands. Nevertheless, the resulting spectra displayed four upward bands at 1192, 1130, and 937 cm^−1^ (Figure S7, Supporting Information), which likely resulted from spontaneous reaction between the electrolyte and soluble LiPSs, possibly formed during a partial self‐discharge of the cell. The integrated area of the bands displayed in the spectra evidenced a similar increasing rate for the bands positioned at 1192, 1130, and 937 cm^−1^, suggesting that those species may have originated from common precursors.

In summary, in situ FTIR spectroscopy provided direct molecular‐level insights into the formation and evolution of the CEI under operating conditions. The appearance of an upward band during the initial discharge indicated the generation of reduced species associated with CEI formation, while characteristic downward bands evidenced concurrent electrolyte decomposition. LiTFSI and LiNO_3_ were identified as the main precursors of the reduced species formed. In situ FTIR spectra also revealed the spontaneous formation of species when the cell was held at OCV, likely attributed to reactions between electrolyte species and soluble LiPSs, with no evidence of electrolyte decomposition under these conditions. Despite these advances and the successful observation of electrolyte solvents and salts consumed during the initial cycle, it remains challenging to identify the chemical composition of the CEI based on in situ FTIR results.

Nevertheless, these findings highlight the correlation between electrolyte decomposition and the CEI mechanism, providing fundamental insights into its behavior. The structural changes revealed by in situ FTIR spectroscopy complement EIS results detailed in section 3.1, which previously indicated changes in the interfacial layers after their establishment (after 5 CVs) based on variations in *τ* and *n*. Despite the limitations, the results presented in this section confirm the effectiveness of the SEC employed for studying the CEI at the transmittance mode, which is versatile and does not require additional accessories. To complement these results and provide deeper insight into the chemical composition of the CEI, synchrotron‐based XAS at the sulfur K‐edge was employed to investigate sulfur species involved in its formation.

### 3.3 XAS Measurements of Post‐Cycled S@SiOC Electrodes

**Figure 6:**
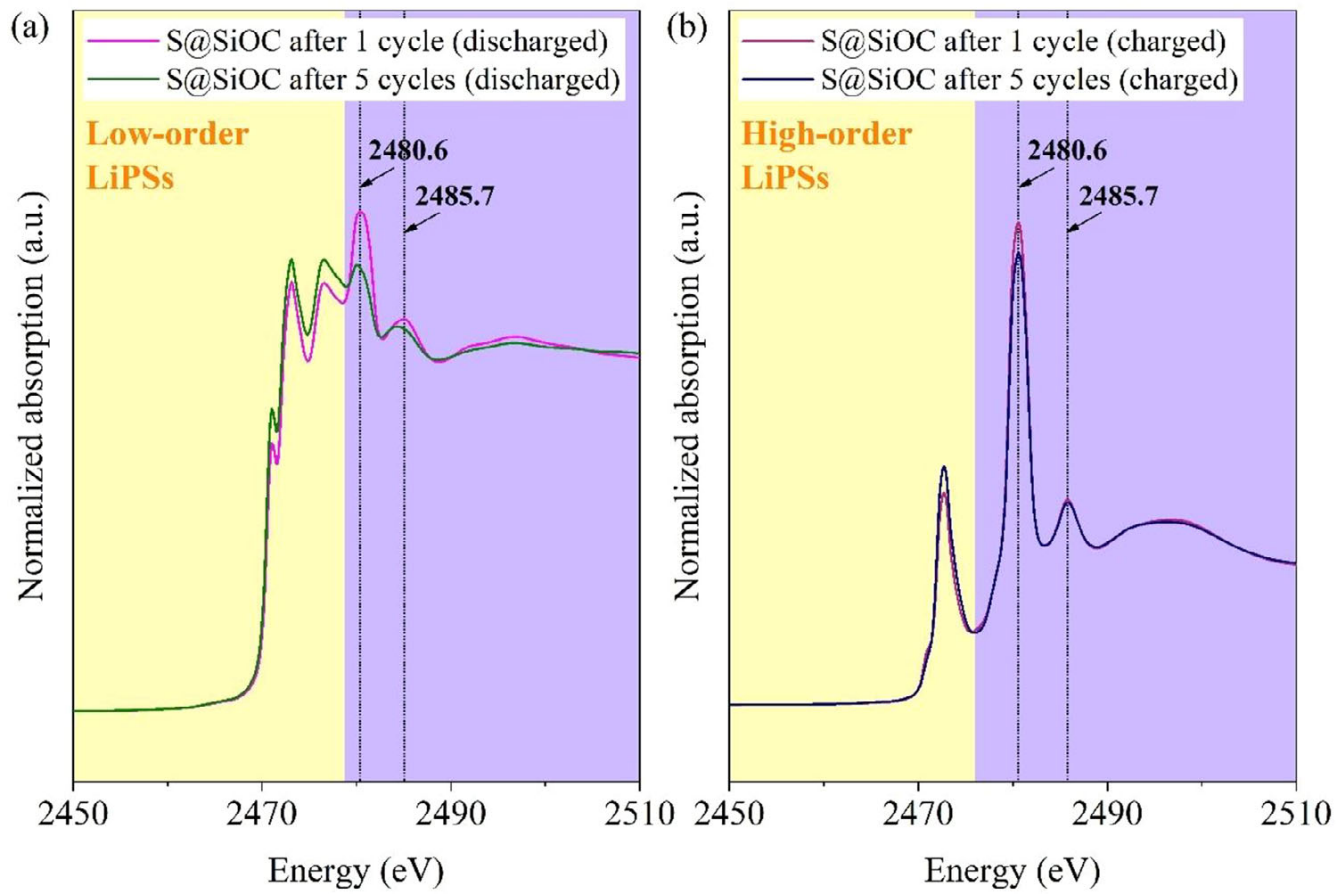
Ex situ measurements of the post‐cycled S@SiOC electrodes after one and five cycles, at the a) fully discharged and b) fully charged states.

To understand changes in the oxidation states of sulfur‐containing species, the S@SiOC electrodes were investigated by synchrotron XANES measurements at the sulfur K‐edge. Ex situ measurements were conducted on pristine and post‐cycled S@SiOC electrodes after one and five cycles at the fully discharged and fully charged states, after cycling at a gravimetric current of 167.5 mA g_s_^−1^. The resulting XANES spectrum of the pristine S@SiOC electrode matched the XANES spectrum of commercial S_8_ (Sigma–Aldrich), confirming the presence of elemental sulfur in the electrode (see Figure S8, Supporting Information). The XANES spectra of the post‐cycled electrodes exhibited additional peaks that were not displayed in the spectrum of the pristine electrode. These additional features include peaks attributed to intermediate LiPSs, positioned within the yellow‐shaded region of **Figure** **6**.

For the post‐cycled electrodes at the discharged state (Figure 6a), the XANES spectra revealed peaks at ≈ 2471.0, 2473.1, and 2476.6 eV within the LiPSs region (yellow‐shaded). These peaks can be attributed to specific intermediate LiPSs, as reported by Pascal et al.^[^ ^37^ ^]^ In the work conducted by Pascal et al.,^[^ ^37^ ^]^ XANES spectra were obtained for different orders of LiPSs using density functional theory (DFT) calculations within the excited electron and core‐hole (XCH). The peaks centered at ≈2471 and 2473.1 eV are similar to those previously observed in the DFT‐calculated spectrum of Li_2_S_4_, while the peak positioned at ≈2476.6 eV is similar to one peak observed in the spectrum of Li_2_S, as reported elsewhere.^[^ ^38^ ^]^ Thus, the resulting XANES spectra indicate the coexistence of soluble Li_2_S_4_ and insoluble Li_2_S on the discharged S@SiOC cathodes.

Furthermore, the XANES spectra of the post‐cycled electrodes at the charged state (Figure 6b) displayed a peak positioned at 2472.7 eV, preceded by a shoulder centered at 2470.7 eV within the LiPSs region (yellow‐shaded), matching the DFT‐simulated spectra of high‐order LiPSs (L_2_S_n_, 7 ≤ n ≤ 8) previously reported by Pascal et al.,^[^ ^37^ ^]^ confirming the oxidation of low‐order LiPSs (e.g., Li_2_S_4_) to high‐order LiPSs (e.g., L_2_S_n_, 7 ≤ n ≤ 8).

Additionally, the XANES spectra of the post‐cycled electrodes revealed additional features beyond the LiPSs region (purple‐shaded), attributed to sulfur‐containing species present in the electrolyte and species that likely composed the CEI. The XANES spectrum of the dried electrolyte on a glass fiber membrane was compared with the XANES spectrum of LiTFSI salt (Sigma–Aldrich), and the resulting spectra showed similar peaks, confirming that the peaks observed in the spectrum of the glass fiber membrane originated from the LiTFSI salt (see Figure S9, Supporting Information).

The XANES spectra of the discharged electrodes showed two peaks, positioned at 2480.6 and 2485.7 eV, attributed to the SO_2_CF_3_ group from TFSI^–^ (of LiTFSI), as confirmed in Figure S10a (Supporting Information). The spectra of the discharged S@SiOC electrodes displayed a lower intensity for the sulfonyl SO_2_CF_3_ group from TFSI^–^, compared to the spectrum of the electrolyte (Figure S10a, Supporting Information), indicating its decomposition during the discharge process for the formation of reduced species.

However, the post‐cycled S@SiOC electrodes at the charged state exhibited a strong LiTFSI signal (Figure 6b; Figure S10b, Supporting Information), suggesting the presence of unreacted LiTFSI. Nevertheless, the S@SiOC electrode evidenced a slight *shoulder* preceding the peak attributed to TFSI^–^ at 2480.6 eV, previously attributed to SO_3_^–^,^[^ ^18^ ^]^ and consistent with the formation of Li_2_SO_3_, indicating its presence in the CEI. These XANES results complement in situ FTIR spectroscopy results (section 3.2), providing additional insights into the sulfur‐containing species that composed the CEI. Furthermore, previous studies have proposed the formation of Li_2_SO_3_ in LSBs,^[^ ^34^, ^35^ ^]^ as detailed in Equations (2)–(6) in Section 3.2.

In summary, XANES measurements at the sulfur K‐edge indicated the formation of sulfur‐containing species that composed the CEI. These findings align with the in situ FTIR results (Section 3.2), which revealed the formation of the CEI alongside the consumption of electrolyte components. Together, these complementary techniques provide molecular‐level insights that support deeper understanding and the rational design of electrode‐electrolyte interphases.

## 4 Conclusion

This work provides insights into the formation and evolution of the CEI in LSBs, which is an interfacial layer that influences the electrochemical stability of LSBs but has been rarely studied. EIS indicated changes attributed to the stabilization of the CEI and the SEI, formed on the cathode and anode, respectively, evidenced by variations in the *τ* and *n*. Furthermore, the electrolyte consumption and the formation of species were evidenced by background‐subtracted in situ FTIR spectroscopy, indicating the formation of the CEI. The contribution of LiNO_3_ has also been confirmed by in situ FTIR spectroscopy, as the resulting spectra for a LiNO_3_‐containing electrolyte evidenced the formation of additional species compared to the results for a *LiNO_3_‐free* electrolyte. Moreover, XANES results at the sulfur K‐edge of post‐cycled S@SiOC electrodes indicated the formation of sulfur‐containing species that composed the CEI. The multi‐technique approach employed in this study provided insights into the CEI, revealing its formation, partial dissolution, and subsequent stabilization. Based on the electrochemical behavior of the LSB system investigated in this work, the insights obtained regarding the CEI are expected to be analogous to other sulfur cathodes exhibiting comparable electrochemical performance. However, the CEI on LSBs still faces challenges, such as its rupture and uneven Li_2_S deposition, which require further investigation. Therefore, the combination of EIS, background‐subtracted in situ FTIR, and XANES measurements can support the design of new electrode and electrolyte materials, including sulfur hosts and electrolyte additives, enabling optimization of the CEI stability.

## Conflict of Interest

The authors declare no conflict of interest.

## Supporting information



Supporting Information

## Data Availability

The data that support the findings of this study are available from the corresponding author upon reasonable request.
