# Supplementary material for: Unveiling the Formation and Evolution of the Cathode–Electrolyte Interphase in Lithium–Sulfur Batteries
Source: Adv Sci (Weinh). 2025 Dec 23;13(9):e18282. doi: 10.1002/advs.202518282 (PMC12904020; doi:10.1002/advs.202518282)
Supplement: Supplementary file 1 — Supporting Information [file ADVS-13-e18282-s001.docx]

**Supplementary information for:**

Murilo Machado Amaral^1, 2, 3^, Otavio Jovino Marques^3^, André Navarro de Miranda^4^, Aline Carlos Oliveira^5^, Gustavo Doubek^4^, Gurpreet Singh^2^, Hudson Zanin^1, *^, Renato Garcia Freitas^5^, Johanna Nelson Weker^3, *^, Pablo Sebastian Fernandez^6, *^

^1^ Advanced Energy Storage Division, Center for Innovation on New Energies, School of Electrical Engineering and Computer, University of Campinas (UNICAMP), Campinas 13083-852, SP, Brazil

^2^ Mechanical and Nuclear Engineering Department, Kansas State University, Manhattan, KS 66506, United States of America

^3^ Stanford Synchrotron Radiation Lightsource, SLAC National Accelerator Laboratory, Menlo Park, CA 94025, United States of America

^4^ Advanced Energy Storage Division, Center for Innovation on New Energies (CINE), Laboratory of Advanced Batteries, School of Chemical Engineering, University of Campinas (UNICAMP), Campinas 13083-852, SP, Brazil

^5^ Institute of Physics & Institute of Chemistry, Laboratory of Computational Materials, Federal University of Mato Grosso, Cuiabá 78060-900, MT, Brazil

^6^ Center for Innovation on New Energies and Chemistry Institute, University of Campinas (UNICAMP), Campinas 13083-852, SP, Brazil

^*^Corresponding authors

E-mail: [pablosf@unicamp.br](mailto:pablosf@unicamp.br); [jlnelson@slac.stanford.edu](mailto:jlnelson@slac.stanford.edu); [hzanin@unicamp.br](mailto:hzanin@unicamp.br)

**1. Electrochemical characterization**

**1.1. Cyclic voltammetry**

The electrochemical behavior of the S@SiOC electrode was investigated using cyclic voltammetry (CV). The initial scan started from the open circuit voltage (OCV) of the LSB cell (i.e., *≈* 2.38 V vs. Li^+^/Li^0^), followed by subsequent CV scans at a voltage window of 1.7 – 2.7 V vs. Li^+^/Li^0^. The CV curves exhibited distinct redox peaks associated with electrochemical reactions during cathodic and anodic sweeps. During the cathodic sweep, two redox peaks (i.e., C_1_ and C_2_) were observed, attributed to the reduction of elemental sulfur (S_8_) to intermediate lithium polysulfides (LiPSs), and the subsequent reduction of intermediate LiPSs to lithium sulfide (Li_2_S) and lithium disulfide (Li_2_S_2_). Furthermore, the CV curves displayed redox peaks during the anodic sweep (i.e., A_1_ and A_2_), attributed to the oxidation of Li_2_S and Li_2_S_2_ to intermediate LiPSs, and their subsequent oxidation to high-order LiPSs and S_8_ [1–3].


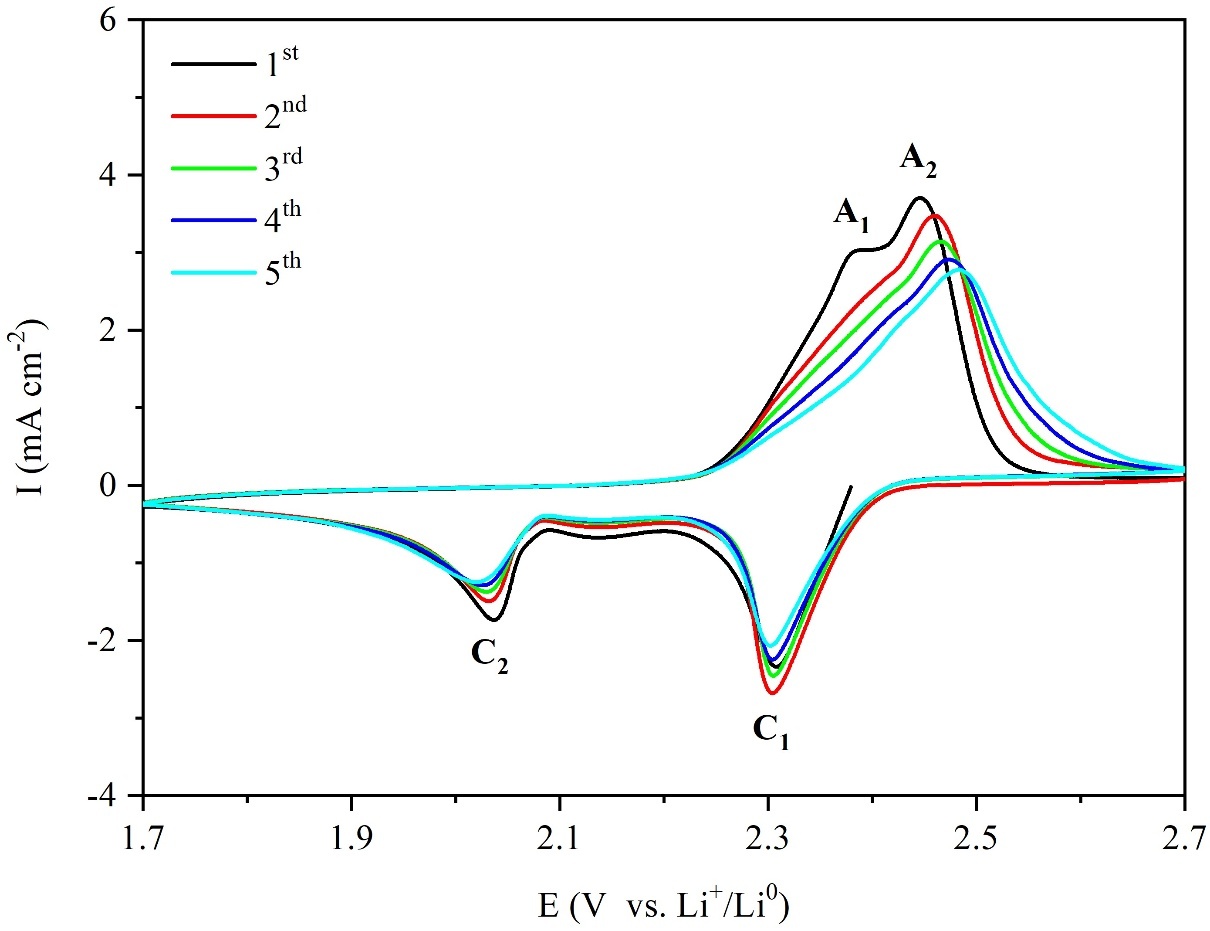


**Figure S1.** Cyclic voltammetry of the LSB cell comprising the S@SiOC as the cathode, after five cycles, during the (*i*) discharging and (*ii*) charging, displaying redox peaks attributed to the reduction and oxidation of LiPSs. Experiments were conducted at 25 ^º^C and *v* = 0.01 mV s^-1^. Inset: Integrated area ratio C_2_/C_1_ along five voltammetric cycles. Positive electrode: S@SiOC. Negative electrode: lithium metal (Li^0^). Electrolyte: 1 mol dm^-3^ LiTFSI in DOL/DME (1:1, v/v) with 1 wt% LiNO_3_. Measurements were performed using a two-electrode CR2032-type cell.

**1.2. Electrochemical impedance spectroscopy**

Among the elements that comprise the *eqvcrts*, the constant-phase element (CPE) represents non-ideal capacitive behavior. The impedance of a CPE is detailed in equation S1, where *n* is a real number between 0 and 1 known as the CPE exponent, *Q* is the magnitude expressed in F cm^-2^ s*^n^*^-1^, *i* is the imaginary unit (*i*^2^ = -1), and *w* is the angular frequency (*w* = 2π*f*) [4,5].

$Z_{CPE}=\frac{1}{Q\left( iw \right)^{n}}$ Equation S1

The total impedance (i.e., $Z_{total}^{eqvcrt-i}$) of *eqvcrt-i*, *eqvcrt-ii*, and *eqvcrt-iii* are detailed in equations S2, S3, and S4, respectively.

$$Z_{total}^{eqvcrt-i}=\frac{1}{Q_{cathode}\left( iw \right)^{n_{cathode}}+\frac{1}{r_{cathode-ct}+\frac{r_{ion}\tanh\left( \sqrt{iw\tau_{s}} \right)}{\sqrt{iw\tau_{s}}}}}+r_{esr}+\frac{1}{Q_{anode}\left( iw \right)^{n_{anode}}+\frac{Q_{str}}{Q_{str}r_{str}+\left( iw \right)^{{-n}_{str}}}}$$

(Equation S2)

$$Z_{total}^{eqvcrt-ii}=\frac{1}{Q_{cathode}\left( iw \right)^{n_{cathode}}+\frac{Q_{str}}{Q_{str}r_{cathode-ct}+\left( iw \right)^{{-n}_{str}}}}+r_{esr}+\frac{r_{anode-ct}}{1+Q_{anode}r_{anode-ct}\left( iw \right)^{n_{anode}}}$$

(Equation S3)

$Z_{total}^{eqvcrt-iii}=\frac{r_{cathode-ct}}{1+Q_{cathode-int}r_{cathode-ct}\left( iw \right)^{n_{cathode-int}}}+\frac{r_{cathode-cei}}{1+Q_{cathode-ext}r_{cathode-cei}\left( iw \right)^{n_{cathode-ext}}}+r_{esr}+\frac{r_{anode-sei}}{1+Q_{anode-ext}r_{anode-sei}\left( iw \right)^{n_{anode-sei}}}+\frac{r_{anode-ct}}{1+Q_{anode-int}r_{anode-ct}\left( iw \right)^{n_{anode-int}}}$

(Equation S4)

**Table S1.** Equivalent circuit (eqvcrt) fitting results from EIS data. The eqvcrts used are indicated by colors, purple: *eqvcrt-i*, green: *eqvcrt-ii*, and blue: *eqvcrt-iii*.

| Discharge | | | | | | | | | | | | | | | | | | | | | | | | | | | | |
| --- | --- | --- | --- | --- | --- | --- | --- | --- | --- | --- | --- | --- | --- | --- | --- | --- | --- | --- | --- | --- | --- | --- | --- | --- | --- | --- | --- | --- |
| E (V) | | **Q_cathode-T_** | **Q_cathode-P_** | | **R_ct_^1^** | | **Q_str-T_** | | **Q_str-P_** | | **R_esr_** | | **Q_anode-T_** | | **Q_anode-P_** | | **R_ct_^2^** | | **Ws-R** | | **Ws-T** | | **Ws-P** | |  | |  |  |
| 2.36 | | 3.39E-06 | 0.93798 | | 3.413 | | 0.23546 | | 0.85581 | | 1.745 | | 5.25E-04 | | 0.91773 | | 11.2 | | 12.13 | | 0.0026611 | | 0.20923 | |  | |  |  |
| 2.34 | | 4.05E-06 | 0.94175 | | 3.521 | | 0.23371 | | 0.7666 | | 1.754 | | 7.01E-04 | | 0.92173 | | 11.42 | | 14.24 | | 0.0067895 | | 0.18443 | |  | |  |  |
| 2.29 | | 4.41E-06 | 0.94173 | | 3.929 | | 0.073432 | | 0.7512 | | 1.745 | | 1.70E-03 | | 0.92524 | | 14.14 | | 24.56 | | 0.49831 | | 0.13064 | |  | |  |  |
| 2.27 | | 4.90E-06 | 0.94427 | | 4.587 | | 0.054973 | | 0.7219 | | 1.738 | | 1.54E-03 | | 0.92389 | | 19.09 | | 24.37 | | 0.56404 | | 0.12554 | |  | |  |  |
| 2.23 | | 5.20E-06 | 0.94294 | | 6.089 | | 0.069986 | | 0.7051 | | 1.742 | | 1.13E-03 | | 0.92573 | | 32.64 | | 16.83 | | 0.045189 | | 0.14779 | |  | |  |  |
| 2.05 | | 8.90E-06 | 0.94534 | | 6.893 | | 0.058986 | | 0.7017 | | 1.766 | | 3.54E-03 | | 0.97752 | | 36.9 | | 39.55 | | 104.7 | | 0.12392 | |  | |  |  |
|  | | **Q_cathode-T_** | **Q_cathode-P_** | | **R_ct_^1^** | | **Q_str-T_** | | **Q_str-P_** | | **R_esr_** | | **Q_anode-T_** | | **Q_anode-P_** | | **R_ct_^2^** | |  | |  | |  | |  | |  |  |
| 1.98 | | 1.10E-05 | 0.81813 | | 11.25 | | 0.011013 | | 0.66278 | | 1.684 | | 6.64E-04 | | 0.97686 | | 498.3 | |  | |  | |  | |  | |  |  |
| 1.7 | | 1.04E-05 | 0.80769 | | 12.4 | | 0.0054945 | | 0.69527 | | 1.77 | | 7.07E-04 | | 0.9924 | | 1178 | |  | |  | |  | |  | |  |  |
| Charge | | | | | | | | | | | | | | | | | | | | | | | | | | | | |
|  | **Q_cathode-T_** | | | **Q_cathode-P_** | | **R_ct_^1^** | | **Q_str-T_** | | **Q_str-P_** | | **R_esr_** | | **Q_anode-T_** | | **Q_anode-P_** | | **R_ct_^2^** | |  | |  | |  | |  | |  |
| 2.11 | 1.22E-04 | | | 0.70221 | | 24.24 | | 0.0032452 | | 0.60857 | | 1.753 | | 0.001851226 | | 0.9883 | | 3240 | |  | |  | |  | |  | |  |
| 2.2 | 3.45E-05 | | | 0.74788 | | 16.37 | | 0.00247 | | 0.65424 | | 1.604 | | 0.0015399 | | 0.98283 | | 741.4 | |  | |  | |  | |  | |  |
|  | **Q_cathode-T_** | | | **Q_cathode-P_** | | **R_ct_^1^** | | **Q_str-T_** | | **Q_str-P_** | | **R_esr_** | | **Q_anode-T_** | | **Q_anode-P_** | | **R_ct_^2^** | | **Ws-R** | | **Ws-T** | | **Ws-P** | |  | |  |
| 2.36 | 6.74E-06 | | | 0.85408 | | 14.6 | | 0.014191 | | 0.913 | | 1.757 | | 0.001812066 | | 0.77361 | | 96.76 | | 24.39 | | 0.0031838 | | 0.30976 | |  | |  |
| 2.39 | 2.56E-06 | | | 0.93562 | | 8.105 | | 0.37597 | | 0.61032 | | 1.697 | | 0.001056511 | | 0.70944 | | 12.79 | | 12.97 | | 0.0017929 | | 0.24539 | |  | |  |
| 2.44 | 2.31E-06 | | | 0.94198 | | 5.824 | | 0.2039 | | 0.63739 | | 1.64 | | 0.170292683 | | 0.68133 | | 23.46 | | 28.98 | | 0.27928 | | 0.16773 | |  | |  |
|  | **Q_cathode-int-T_** | | | **Q_cathode-int-P_** | | **R_ct_^1^** | | **Q_cathode-ext-T_** | | **Q_cathode-ext-P_** | | **R_CEI_** | | **R_esr_** | | **Q_anode-int-T_** | | **Q_anode-int-P_** | | **R_ct_^2^** | | **Q_anode-ext-T_** | | **Q_anode-ext-P_** | | **R_SEI_** | |  |
| 2.51 | 3.07E-06 | | | 0.97549 | | 4.368 | | 1.16E-05 | | 0.89604 | | 7.027 | | 1.562 | | 0.00069772 | | 0.60384 | | 442.4 | | 0.001361393 | | 0.85267 | | 7.698 | |  |
| 2.57 | 2.94E-06 | | | 0.97697 | | 4.706 | | 1.37E-05 | | 0.88967 | | 6.875 | | 1.572 | | 0.00069607 | | 0.58243 | | 1059 | | 0.086549254 | | 0.84911 | | 8.517 | |  |
| 2.65 | 2.86E-06 | | | 0.98083 | | 4.746 | | 1.35E-05 | | 0.89043 | | 7.094 | | 1.565 | | 0.00067177 | | 0.5775 | | 2265 | | 0.160603758 | | 0.84892 | | 9.474 | |  |

The statistical parameters of the EIS fitting, particularly Chi-square (χ^2^) and sum of squares (SS), were obtained using the ZView^®^ software from Scribner Associates Inc. The χ^2^ and SS values, for both negative and positive polarization, are displayed in Table S2.

**Table S2.** Chi-square (χ^2^) and sum of squares (SS) values for fitting EIS data obtained during the cell polarization. Experiments were conducted using a two-electrode CR2032-type cell. Cathode: S@SiOC. Anode: lithium metal (Li^0^). Electrolyte: 1.0 mol dm^-3^ LiTFSI in DOL/DME (1:1, v/v) with 1 wt% LiNO_3_.

| Voltage  (V vs. Li^+^/Li^0^) | χ^2^ (Ω²) | SS (Ω²) |
| --- | --- | --- |
| Negative polarization | | |
| 2.36 | 3.683×10^-6^ | 3.241×10^-4^ |
| 2.34 | 5.734×10^-6^ | 5.459×10^-4^ |
| 2.29 | 3.762×10^-6^ | 3.105×10^-4^ |
| 2.27 | 4.149×10^-6^ | 3.651×10^-4^ |
| 2.23 | 4.212×10^-6^ | 3.705×10^-4^ |
| 2.05 | 6.105×10^-6^ | 4.604×10^-4^ |
| 1.98 | 7.481×10^-6^ | 5.910×10^-4^ |
| 1.7 | 3.580×10^-6^ | 3.147×10^-4^ |
| Positive polarization | | |
| 2.11 | 3.532×10^-6^ | 3.285×10^-4^ |
| 2.20 | 6.884×10^-6^ | 6.402×10^-4^ |
| 2.36 | 5.779×10^-6^ | 5.201×10^-4^ |
| 2.39 | 2.296×10^-6^ | 2.066×10^-4^ |
| 2.44 | 6.976×10^-6^ | 6.139×10^-4^ |
| 2.51 | 1.086×10^-6^ | 9.455×10^-4^ |
| 2.57 | 9.178×10^-6^ | 7.984×10^-4^ |
| 2.65 | 1.082×10^-6^ | 9.421×10^-4^ |

**2. Background-subtracted in-situ FTIR spectroscopy**

**
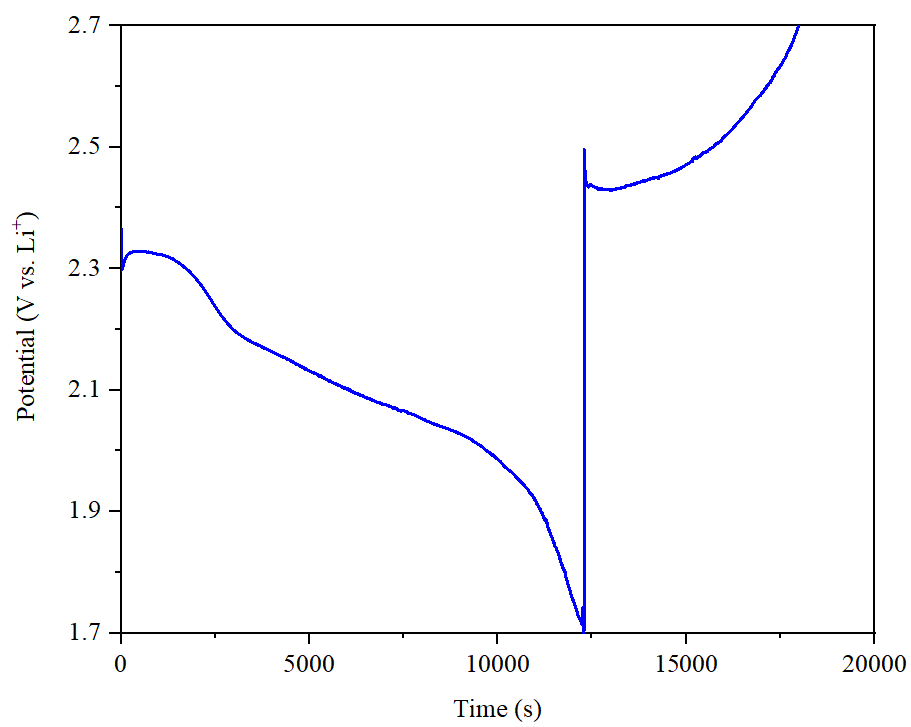
**

**Figure S2.** Charge-discharge profile of the SEC used for in-situ infrared spectroscopy, comprising 1.0 mol dm^-3^ LiTFSI with 1 wt. % LiNO_3_ dissolved in DOL/DME (1:1, v/v) as the electrolyte. Cathode: S@SiOC. Anode: Lithium metal (Li^0^). The galvanostatic charge-discharge experiment has been conducted at 0.2 C (considering 1C = 1675 mAh g_s_^-1^).

**
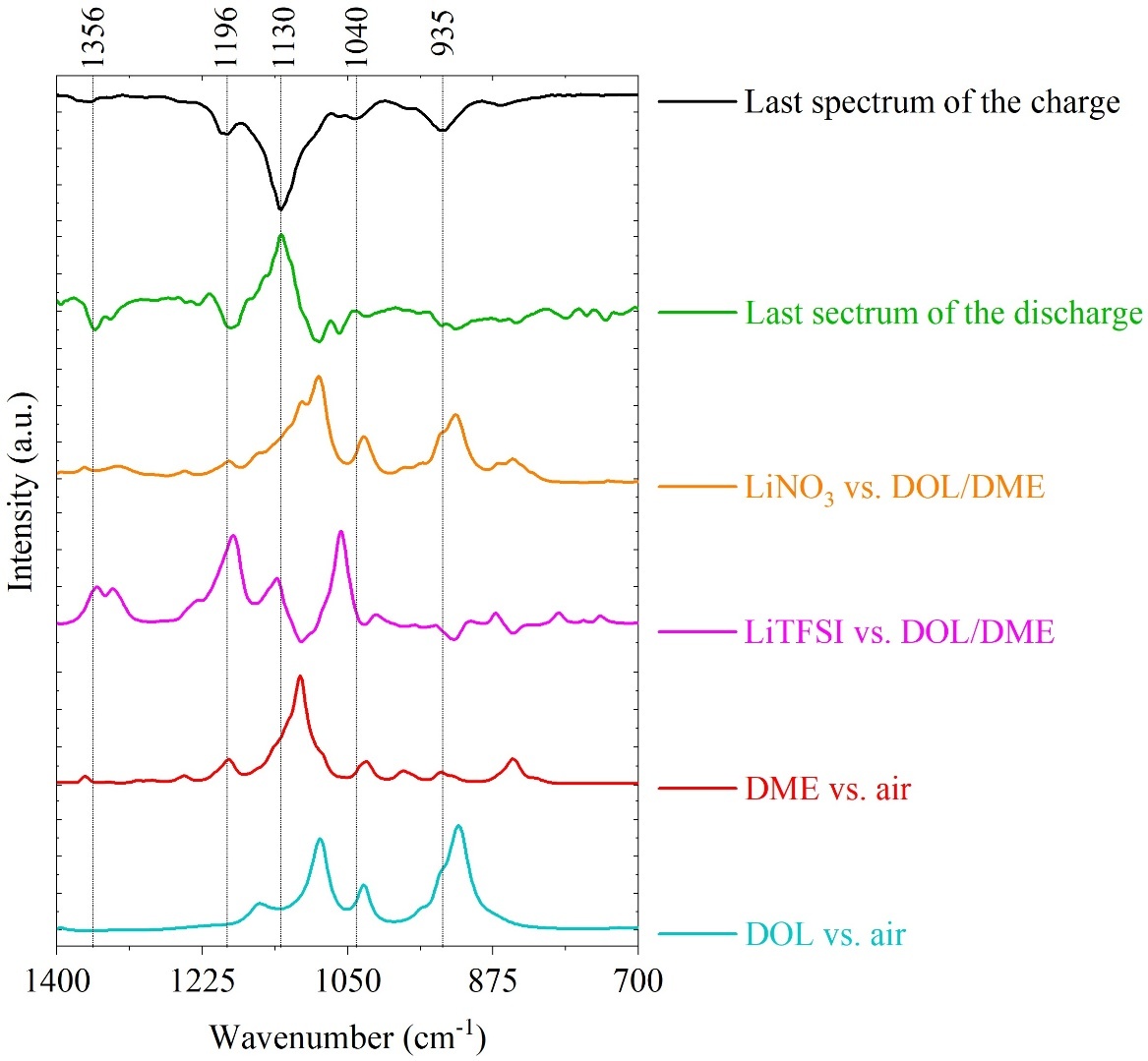
**

**Figure S3.** Comparison of the last spectra of the discharge and charge with the ex-situ spectra of the DOL vs. air, DME vs. air, 7.0 mol dm^-3^ LiTFSI (unsaturated) in DOL/DME (1:1) vs. DOL/DME (1:1), and 0.75 mol dm^-3^ LiNO_3_ (saturated) in DOL/DME (1:1) vs. DOL/DME (1:1).

**Table S3.** Attribution of species based on the standard spectra displayed in Figure 3.

| **Discharging** | **Charging** | **Attribution** |
| --- | --- | --- |
| **Position (cm^-1^)** | |  |
| 1356 (downward) | 1356 (downward) | LiTFSI |
| 1196 (downward) | 1196 (downward) | LiTFSI, LiNO_3_, and DME |
| 1130 (upward) | 1130 (downward) | Reduced species |
| - | 1040 (downward) | LiTFSI |
| - | 935 (downward) | LiNO_3_, DME, and DOL |


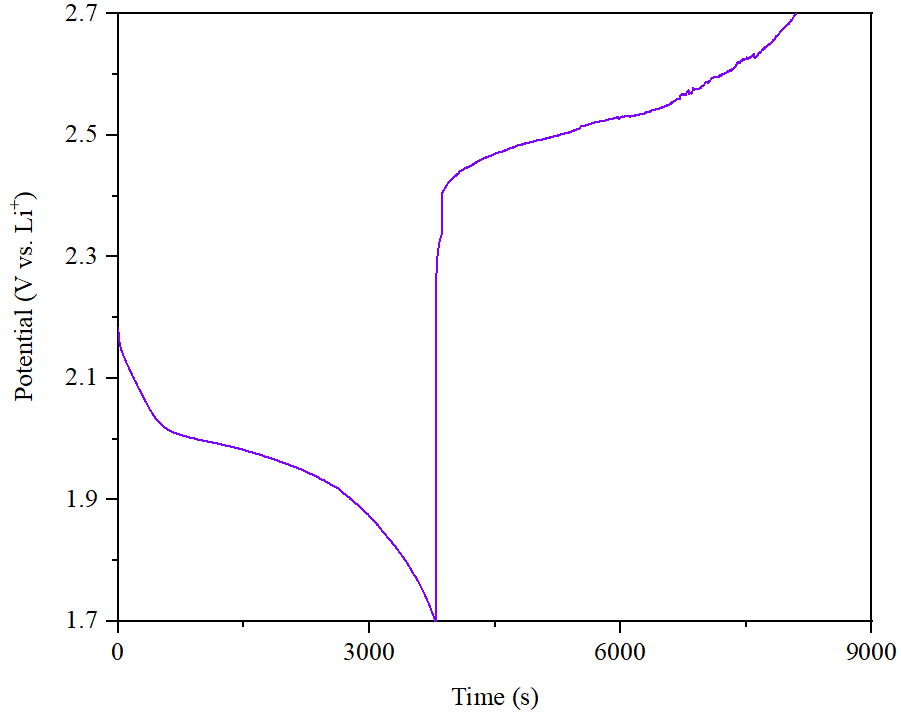


**Figure S4.** Charge-discharge profile of the SEC used for in-situ infrared spectroscopy, comprising 1.0 mol dm^-3^ LiTFSI dissolved in DOL/DME (1:1, v/v) as the electrolyte (LiNO_3_-free). Cathode: S@SiOC. Anode: Lithium metal (Li^0^). The galvanostatic charge-discharge experiment has been conducted at 0.2 C (considering 1C = 1675 mAh g_s_^-1^).

**
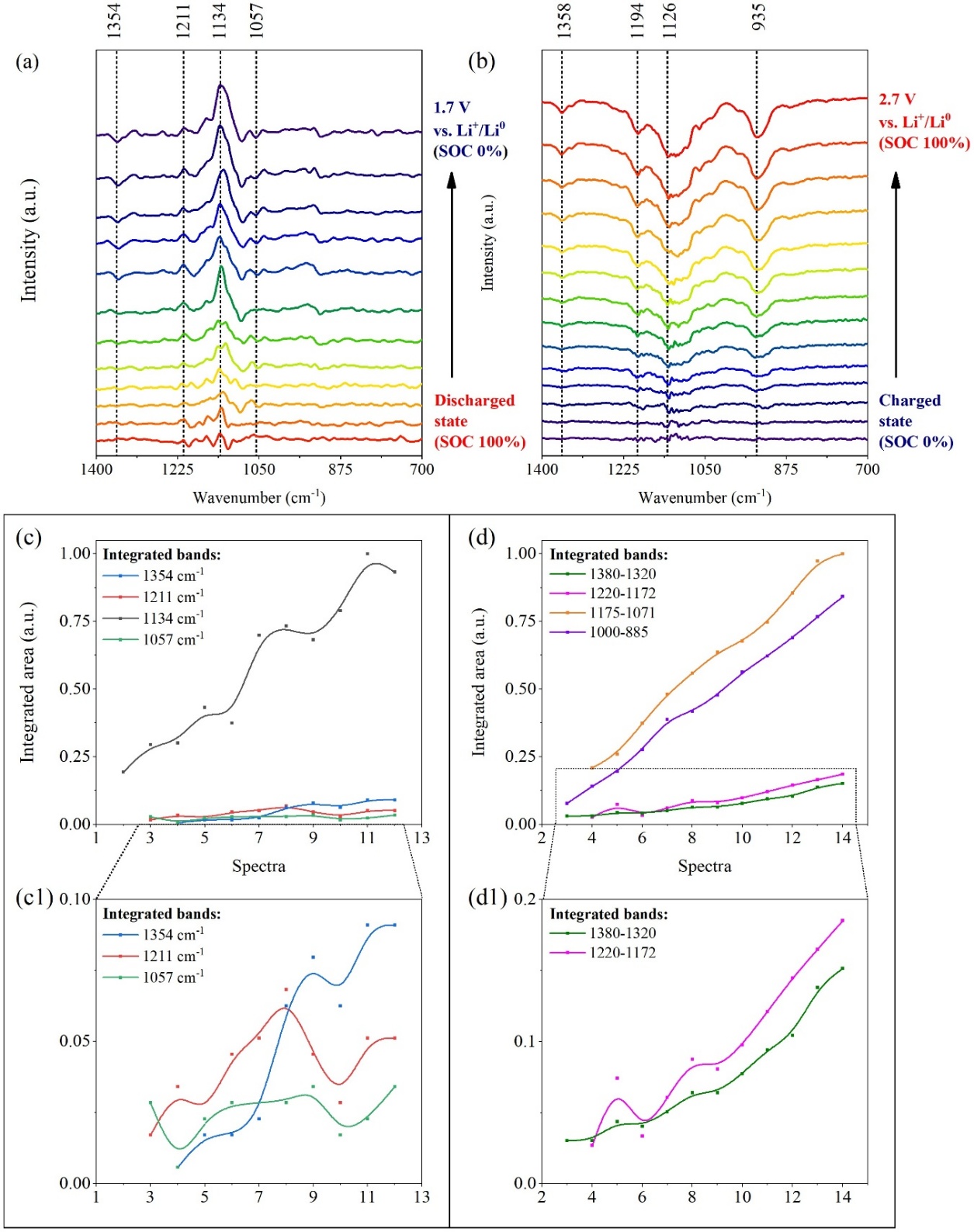
**

**Figure S5.** In-situ FTIR spectra of the LSB cell containing 1.0 mol dm^-3^ LiTFSI in DOL/DME electrolyte (LiNO_3_-free) during the initial (a) discharge and (b) charge. Integrated areas of the most intensive bands displayed during the (c) discharge and (d) charge.

**
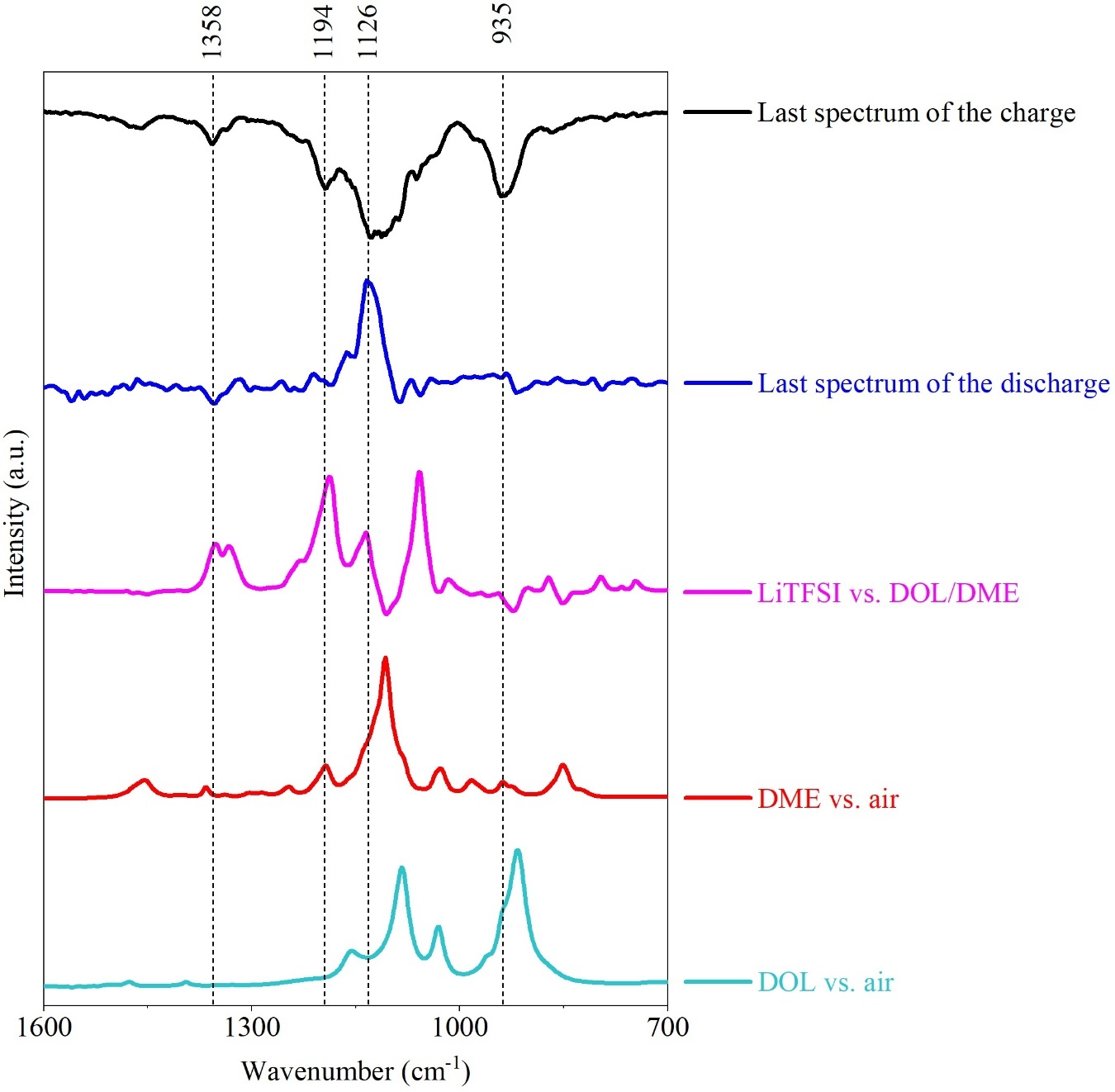
**

**Figure S6.** Comparison of the last spectra of the discharge and charge with the ex-situ spectra of the DOL vs. air, DME vs. air, and 7.0 mol dm^-3^ LiTFSI (unsaturated) in DOL/DME (1:1) vs. DOL/DME (1:1).

**Table S4.** Attribution of species based on the standard spectra displayed in Figure S6.

| **Discharging** | **Charging** | **Attribution** |
| --- | --- | --- |
| **Position (cm^-1^)** | |  |
| 1354 (downward) | 1358 (downward) | LiTFSI |
| 1211 (upward) | - | Reduced species |
| - | 1194 (downward) | LiTFSI and DME |
| 1134 (upward) | 1126 (downward) | Reduced species |
| 1057 (downward) | - | LiTFSI |
| - | 935 (downward) | DME and DOL |


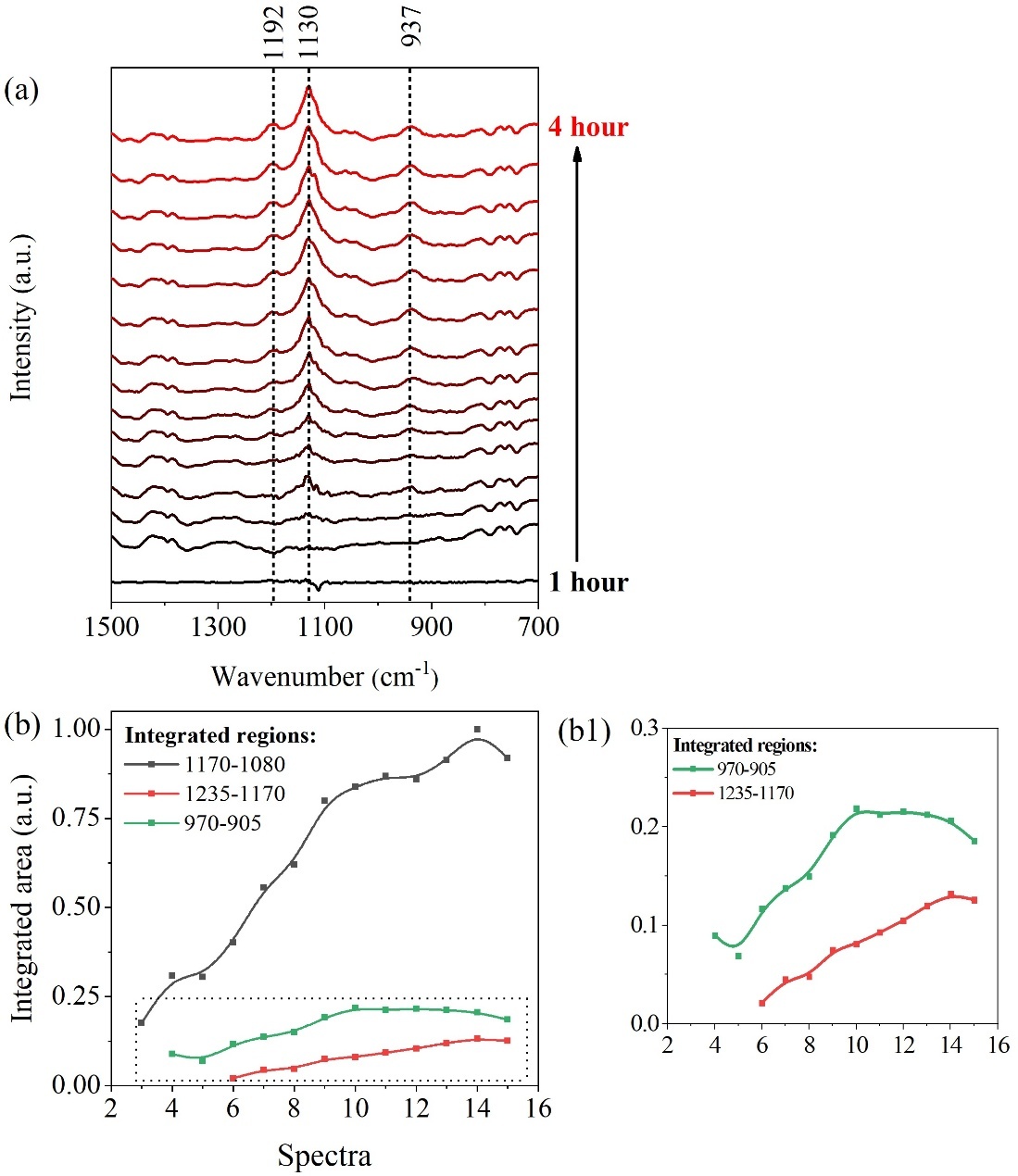


**Figure S7.** (a) FTIR spectra of the LSB cell containing 1.0 mol dm^-3^ LiTFSI in DOL/DME electrolyte (LiNO_3_-free) under the OCP for four hours. (b) Integrated areas of the bands formed during the in-situ FTIR measurement.

**3. Synchrotron X-ray absorption spectroscopy at the sulfur K-edge of post-cycled electrodes**

**
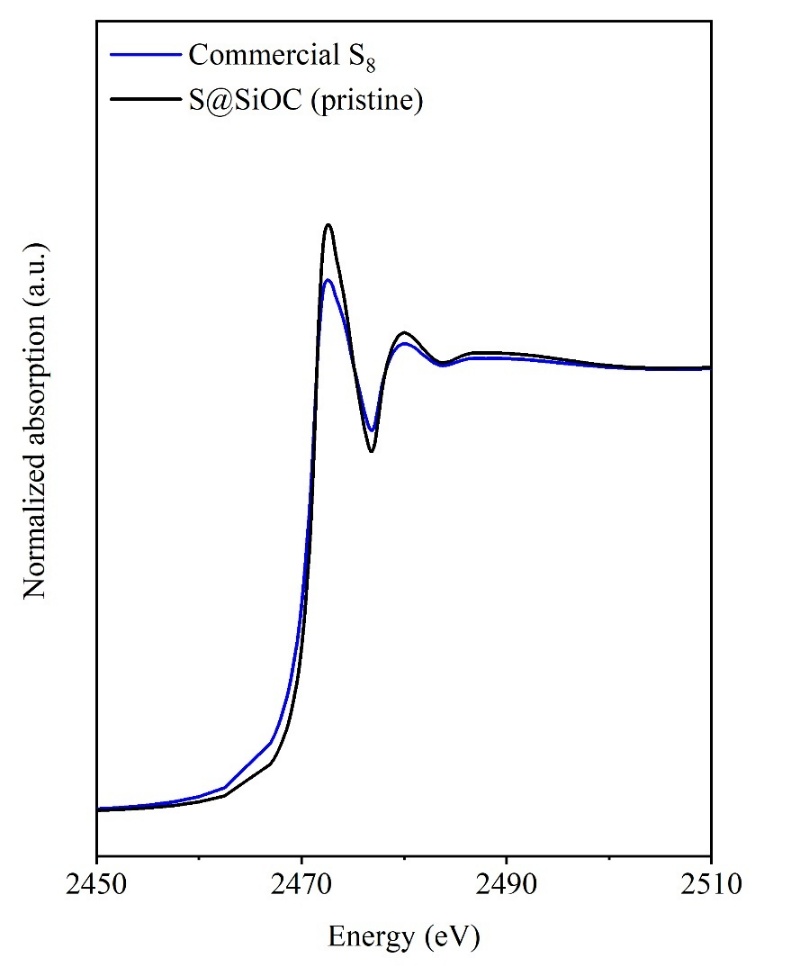
**

**Figure S8.** XANES spectra at the S K-edge for the commercial sulfur powder (Sigma-Aldrich) and pristine S@SiOC electrode.


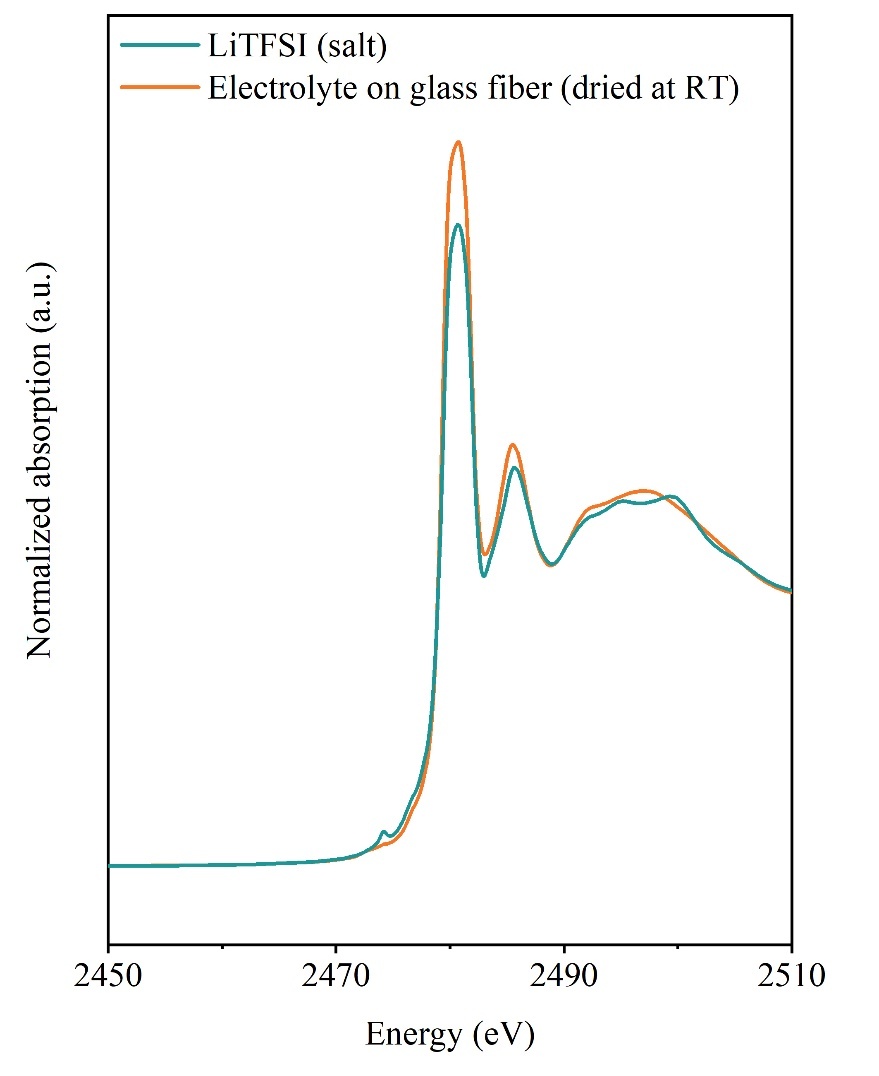


**Figure S9.** XANES spectra at the S K-edge for commercial LiTFSI salt (Sigma-Aldrich) and glass fiber membrane soaked in 1.0 mol dm^-3^ LiTFSI in DOL/DME (1:1, v/v) with 1 wt.% LiNO_3_ electrolyte, followed by drying at room temperature.


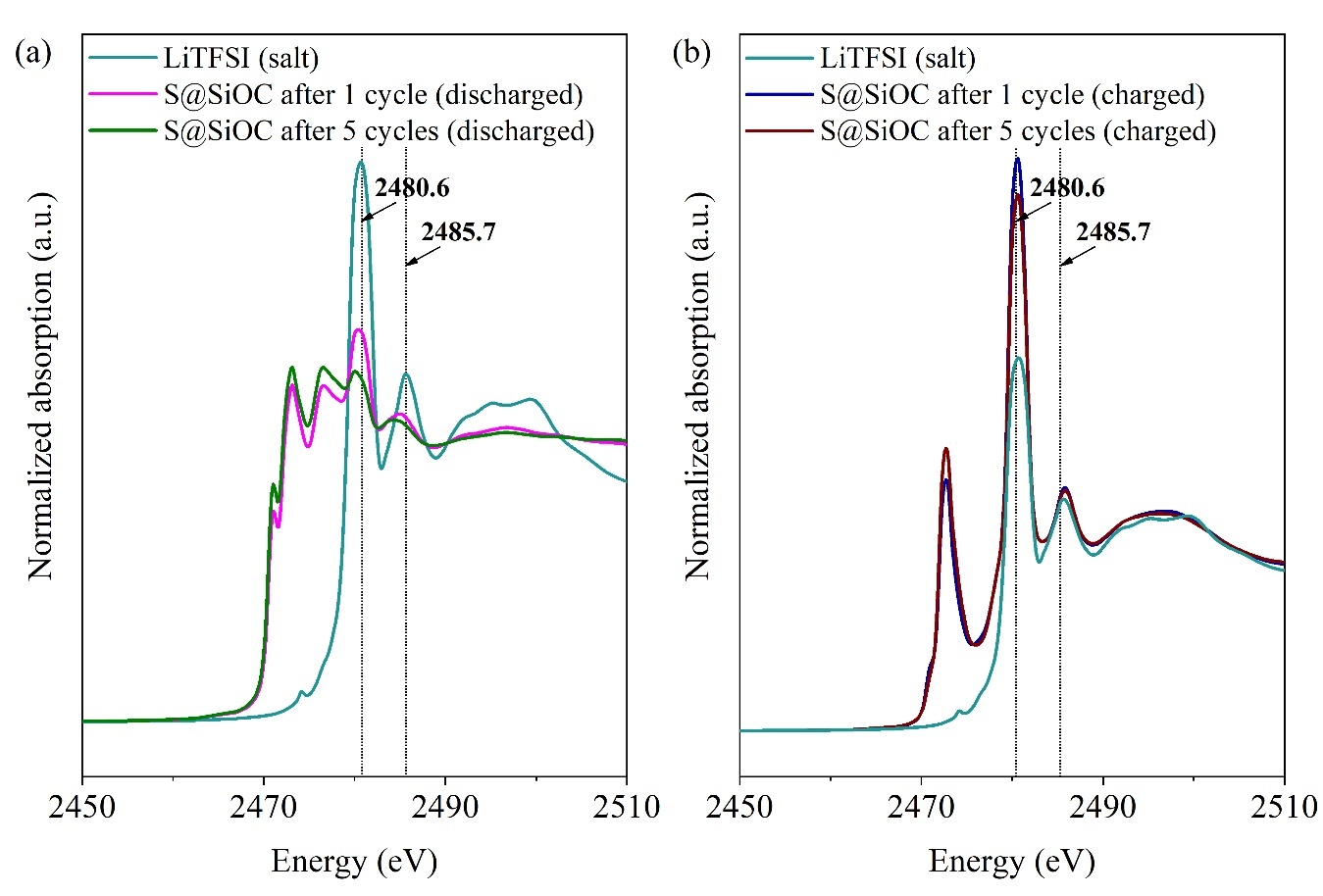


**Figure S10.** Comparison between the spectrum of the post-cycled electrodes at the (a) fully discharged and (b) fully charged states with the spectrum of the commercial LiTFSI salt (Sigma-Aldrich). The spectra displayed peaks positioned at 2480.6 and 2485.7 eV, originating from the SO_2_CF_3_ group of the TFSI^–^ anion.

**References**

[1] Chung, S.-H. and Manthiram, A. (2018). Designing Lithium–Sulfur Batteries with High-Loading Cathodes at a Lean Electrolyte Condition. *ACS Applied Materials & Interfaces*. https://doi.org/10.1021/acsami.8b17393.

[2] Lu, Y. *et al.* (2017). Sulfonic Groups Originated Dual-Functional Interlayer for High Performance Lithium–Sulfur Battery. *ACS Applied Materials & Interfaces*. https://doi.org/10.1021/acsami.7b02142.

[3] Huang, X. *et al.* (2019). Cyclic Voltammetry in Lithium–Sulfur Batteries—Challenges and Opportunities. *Energy Technology*. https://doi.org/10.1002/ente.201801001.

[4] Lasia, A. (2014). *Electrochemical Impedance Spectroscopy and its Applications*, Springer New York, New York, NY.

[5] Jorcin, J.-B. *et al.* (2006). CPE analysis by local electrochemical impedance spectroscopy. *Electrochimica Acta*. https://doi.org/10.1016/j.electacta.2005.02.128.
